# Single-cell atlases link macrophages and CD8^+^ T-cell subpopulations to disease progression and immunotherapy response in urothelial carcinoma

**DOI:** 10.7150/thno.77281

**Published:** 2022-11-14

**Authors:** Yuan Liang, Yezhen Tan, Bao Guan, Bin Guo, Mancheng Xia, Juan Li, Yue Shi, Zihui Yu, Qi Zhang, Di Liu, Xiaopeng Yang, Junfeng Hao, Yanqing Gong, Muhammad Shakeel, Liqun Zhou, Weimin Ci, Xuesong Li

**Affiliations:** 1 CAS Key Laboratory of Genomic and Precision Medicine, Beijing Institute of Genomics, Chinese Academy of Sciences and China National Center for Bioinformation, Beijing 100101, China.; 2 Department of Urology, Peking University First Hospital, Beijing 100034, China.; 3 University of Chinese Academy of Sciences, Beijing 100049, China.; 4 Institute of Urology, Peking University, Beijing 100034, China.; 5 Core Facility for Protein Research, Institute of Biophysics, Chinese Academy of Science, Beijing 100101, China.; 6 National Urological Cancer Center, Beijing Key Laboratory of Urogenital Diseases (Male) Molecular Diagnosis and Treatment Center, Beijing, 100034, China.; 7 Jamil-ur-Rahman Center for Genome Research, Dr. Panjwani Center for Molecular Medicine and Drug Research, ICCBS, University of Karachi, Karachi, Pakistan.; 8 Institute for Stem Cell and Regeneration, Chinese Academy of Sciences, Beijing 100101, China.

**Keywords:** Immunosuppression, Immunotherapy, Single-cell RNA sequencing, Tumor microenvironment, Urothelial carcinoma

## Abstract

**Rationale:** Immune checkpoint inhibitors (ICIs) have revolutionized the management of locally advanced or metastatic urothelial carcinoma. Strikingly, compared to urothelial carcinoma of the bladder (UCB), upper tract urothelial carcinoma (UTUC) has a higher response rate to ICIs. The stratification of patients most likely to benefit from ICI therapy remains a major clinical challenge.

**Methods:** In this study, we performed the first single-cell RNA sequencing (scRNA-seq) study of 13 surgical tissue specimens from 12 patients with UTUC. The key results were validated by the analysis of two independent cohorts with bulk RNA-seq data for UCB (n = 404) and UTUC (n = 158) and one cohort of patients with metastatic urothelial carcinoma (mUC) who were treated with atezolizumab (n = 348).

**Results:** Using scRNA-seq, we observed a higher proportion of tumor-infiltrating immune cells in locally advanced UTUC. Similar prognostically relevant intrinsic basal and luminal-like epithelial subtypes were found in both UTUC and UCB, although UTUC is predominantly of the luminal subtype. We also discovered that immunosuppressive macrophages and exhausted T-cell subpopulations were enriched in the basal subtype and showed enhanced interactions. Furthermore, we developed a gene expression signature (Macro-C3 score) capturing the immunosuppressive macrophages that better predicts outcomes than the currently established subtypes. We also developed a computational method to model immune evasion, and the Macro-C3 score predicted therapeutic response of mUC treated with first-line anti-PD-L1 inhibitors in patients with lower basal scores.

**Conclusions:** Overall, the distinct microenvironment and Macro-C3 score provide an explanation for ICI efficacy in urothelial carcinoma and reveal new candidate regulators of immune evasion, suggesting potential therapeutic targets for improving antitumor immunity in the basal subtype.

## Introduction

Urothelial carcinoma (UC) is the fourth most common tumor type, and upper tract urothelial carcinoma (UTUC) is a distinctly aggressive genitourinary entity of UC. UTUC arises from the urothelial lining of the upper urinary tract, which includes the renal pelvis and ureter 1. Despite the common histologic appearance of urothelial carcinoma of the bladder (UCB), approximately 60% of UTUCs and 15-20% of UCBs are diagnosed as muscle-invasive (MI), which correlates with a worse prognosis than non-muscle-invasive (NMI) cases with an excellent overall prognosis 2. Genomic and epigenetic subtyping of UCB and UTUC, including that completed by our group, has revolutionized the current understanding of UC pathogenesis 3-7. However, even the most recent consensus molecular classification study of UTUC 8 does not provide compelling evidence for molecular pathogenesis and thus lacks the ability to predict the therapeutic response.

Tumor phenotype is represented by cell state measured as RNA expression. Therefore, cancer subtyping based on gene expression profiles can provide valuable insights into cancer biology and important clues for treatment planning. Notably, a previous study identified two intrinsic subtypes of muscle-invasive UCB, termed “luminal” and “basal-like”, which have the characteristics of different stages of urothelial differentiation. Moreover, a 47-gene predictor, bladder cancer analysis of subtypes by gene expression (BASE47), was developed to subtype UCB into prognostic subtypes 9. However, it remains unclear whether these bulk RNA-seq data mask heterogeneity at the single-cell level derived from the presence of both malignant and nonmalignant cells. Moreover, it remains unclear whether similar prognostically relevant intrinsic subtypes exist in UTUC, and thus, our understanding of their relationship to therapeutic response is limited.

Cell state is an integration of inputs from cell-intrinsic (e.g., mutational background, epigenetic state) and cell-extrinsic (e.g., cell-to-cell interactions, tumor microenvironment) sources. Dynamic interactions between the tumor and tumor microenvironment (TME) are essential for cancer cell heterogeneity and therapeutic response 10. In particular, distinct populations of T cells influence tumorigenesis by performing pro- and anti-tumorigenic functions. T cell exhaustion is the main reason why they fail to eliminate tumor cells and various forms of cancer immunotherapy are now effective in re-establishing and promoting T cell anti-tumor immunity 11, 12. By suppressing T cell function, other immune cell types maintain immune equilibrium, such as M2 polarized macrophages and tolerogenic dendritic cells 13. Tumor-associated macrophages (TAMs) account for a significant fraction in human solid malignancies and influence tumor progression by providing nutritional support to malignant cells, now constituting promising targets for novel anticancer agents 14. Advancements in single-cell RNA sequencing (scRNA-seq) have led to the ability to simultaneously evaluate the cell states of both malignant and nonmalignant cells in TME and thus provide unique opportunities to explore how tumor cell subtypes are shaped by cell-intrinsic and cell-extrinsic factors. In this context, we hypothesized that comprehensive profiling of malignant and nonmalignant cells in UTUC at the single-cell level would enable the deconvolution of current molecular subtypes into their constituent parts, which would consequently enable the development of more effective prognostic and predictive tools.

In this study, we performed single-cell RNA sequencing of 13 surgical tissue specimens obtained from 12 patients with UTUC. We observed that MI UTUC had a higher abundance of tumor-infiltrating immune cells than NMI UTUC. We also found that similar prognostically relevant intrinsic basal and luminal-like subtypes exist in UTUC. Notably, we identified distinct subtype-specific immunological phenotypes in both UTUC and UCB and determined their regulation by the tumor microenvironment and their response to immune checkpoint therapy.

## Results

### Single-cell transcriptomic landscape in UTUC

**Figure 1:**
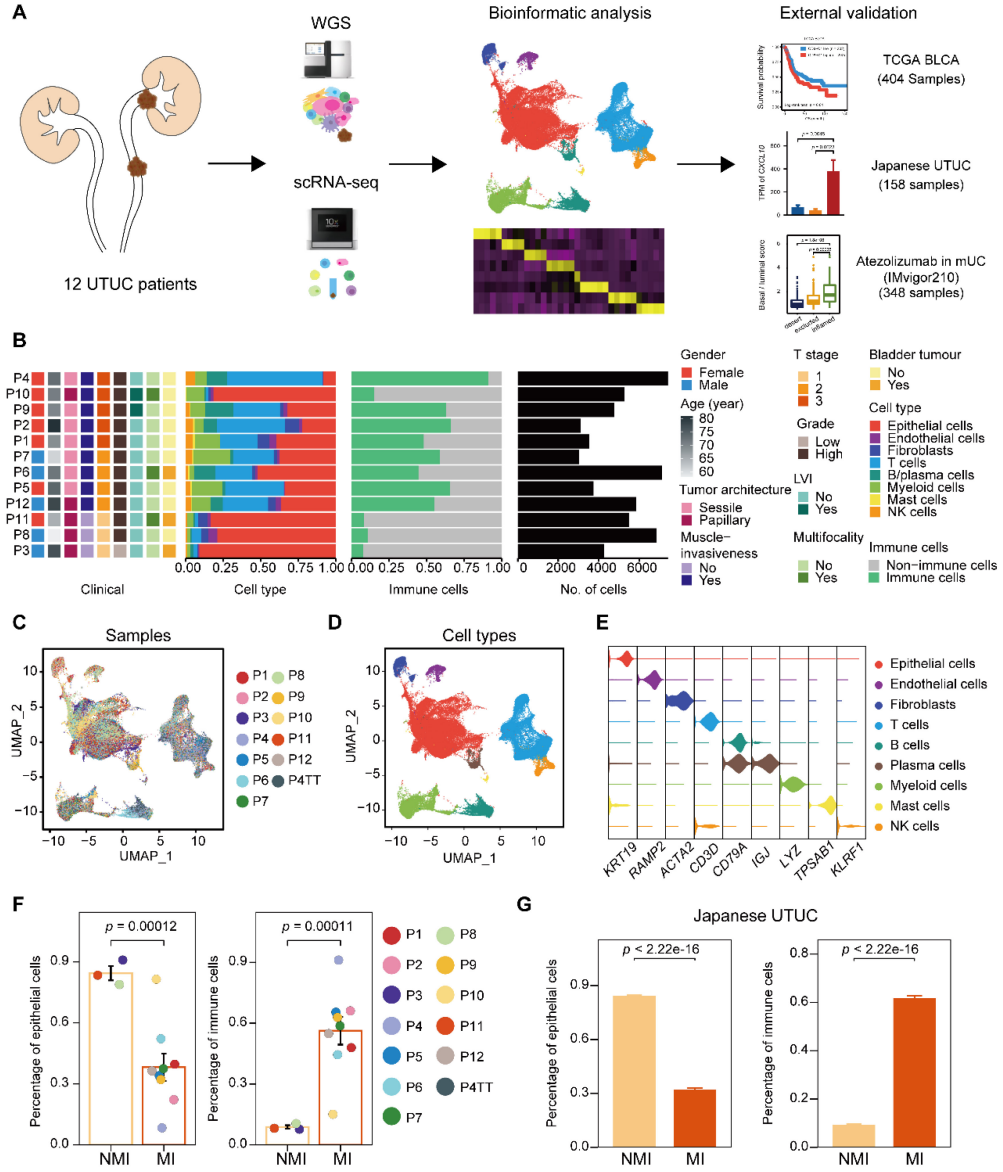
** Single-cell RNA sequencing reveals intertumor heterogeneity of UTUC. (A)** A schematic description of the overall experimental design and data exploration workflow. **(B)** Clinical features of the patients and the composition of cells in the tumor samples. The samples are ordered according to the T category. LVI, lymphovascular invasion. **(C and D)** UMAP of 67,392 cells post-QC and filtering grouped by the sample (C) and major cell type (D). **(E)** Violin plots showing the distribution of expression levels of canonical cell type markers. **(F)** Fractions of epithelial and immune cells in each of the NMI and MI samples. **(G)** Fractions of epithelial and immune cells in each of the NMI and MI samples in the Japanese cohort. In (F) and (G), error bars represent the mean ± standard error of the mean. *P* values were calculated with a *t* test.

To characterize intratumoral heterogeneity, we collected 13 surgical tissue samples, including 12 primary tumors and a paired tumor thrombus, from 12 UTUC patients for scRNA-seq (**Supplementary Table S1**). We also included two cohorts with bulk RNA-seq for UCB and UTUC, and one cohort of atezolizumab-treated patients with metastatic urothelial carcinoma (IMvigor210) 15, 16 for further validation (**Figure 1A**). Quality control of the scRNA-seq data resulted in the inclusion of 67,392 cells (**Supplementary Figure S1A**), which were then integrated into an unbatched dataset -(**Figure 1B & 1C**). Subsequent unsupervised clustering suggested the presence of 22 cell subclusters (**Supplementary Figure S1B & S1C**). Based on gene expression marker, the cells were assigned to nine major cell types, including T cells, myeloid cells, epithelial cells, fibroblasts, endothelial cells, mast cells, B cells, plasma cells, and natural killer (NK) cells (**Figure 1D, 1E & Supplementary Figure S1D**). Although there were few cases, muscle-invasive (MI) tumors (n=3) showed greater enrichment of immune cells and a lower proportion of epithelial cells than nonmuscle-invasive (NMI) tumors (n=9) (**Figure 1F & Supplementary Figure S1E**). Furthermore, we examined a larger cohort of UTUC patients 8 with available bulk transcriptome data and observed significantly increased proportions of immune cell subsets in the MI tumors compared to the NMI tumors (**Figure 1G & Supplementary Figure S1F**). These findings suggest that crosstalk between tumor cells and the immune environment may play a role in shaping muscle invasiveness.

### Intrinsic subtypes of MI UTUC reflect the luminal and basal subtypes of MI UCB

**Figure 2:**
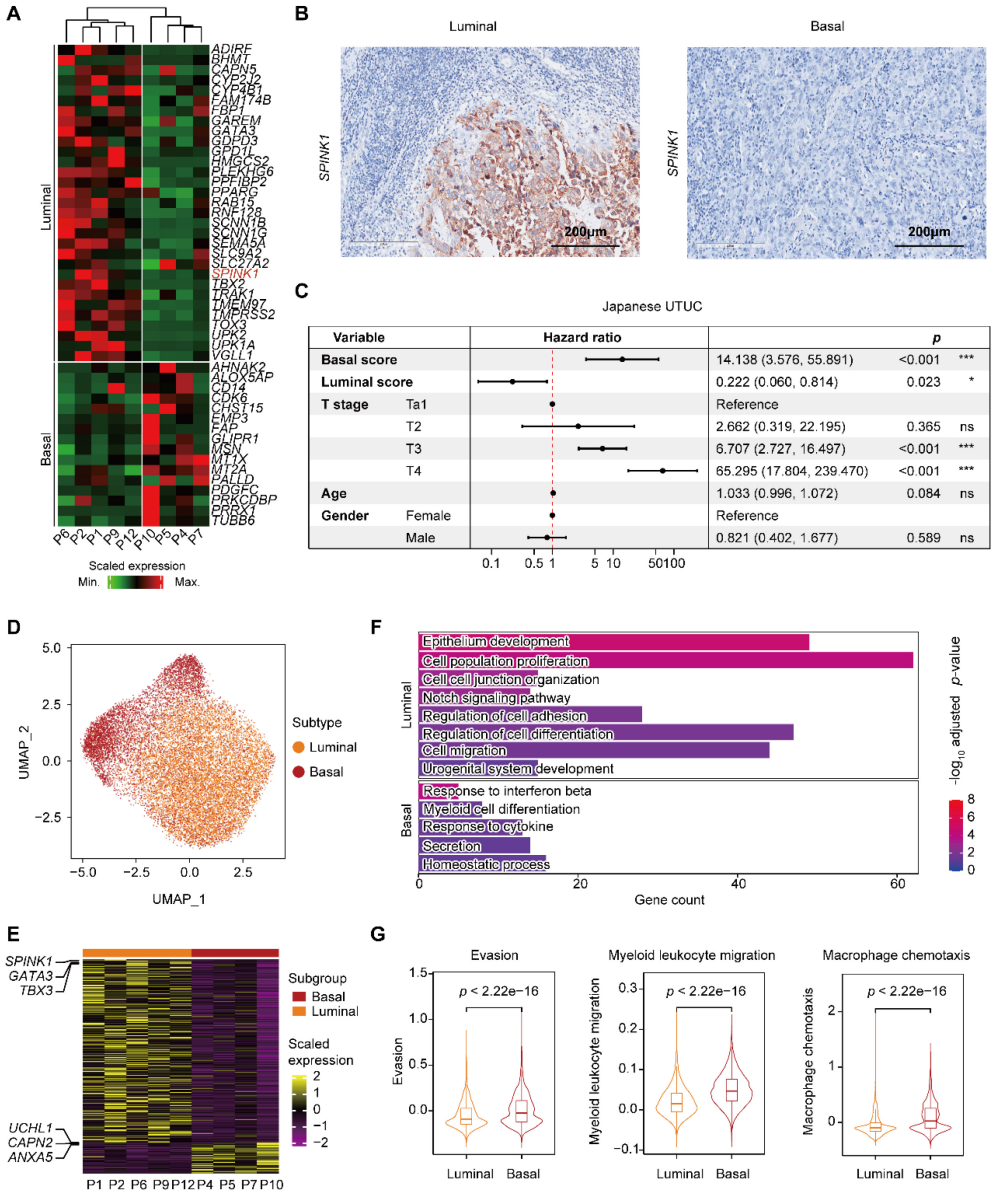
** Classification of MI UTUC into luminal and basal subtypes based on BASE47 gene expression. (A)** Clustering analysis of the MI UTUC samples based on the average expression levels of the BASE47 gene in epithelial cells. The dendrogram is separated into two groups to reflect the distinct expression patterns of the luminal and basal subtypes. **(B)** Representative IHC images of luminal and basal UTUC tissue sections stained for SPINK1. Scale bars correspond to 200 µm. **(C)** Forest plots showing the hazard ratios associated with the luminal score, basal score, and clinical information in univariate Cox proportional hazard models for DSS in the Japanese UTUC cohort. **(D)** UMAP of epithelial cells in luminal and basal UTUC. **(E)** Heatmap showing DEGs between the luminal and basal subtypes based on the average gene expression in epithelial cells. Representative genes are indicated (left). **(F)** Bar plot displaying the statistically significant overrepresentation of GO BP terms in DEGs defined by the UTUC subtypes. **(G)** Violin plots displaying the evasion, myeloid migration, and macrophage chemotaxis scores of tumor cells in the luminal and basal subtypes. The *P* value was calculated by the Wilcoxon test.

To better understand the cellular programs active in cancer cells that may function together with immune cells, we next sought to identify the intrinsic developmental subtypes of the malignant cells. We first confirmed 31,152 malignant cells based on large-scale chromosomal copy number variations (CNVs) in each cell (**Supplementary Figure S2A-C**) inferred by the inferCNV tool 17. Considering the rarity of UTUC, we used the BASE47 panel, a 47-gene predictor defining malignant cells of MI UCB into prognostic subtypes 9, and identified two distinct expression profiles, termed luminal and basal, in the MI UTUC samples (**Figure 2A & Supplementary Table S2**). However, with the limited number of cases, all three NMI UTUCs were classified as the luminal subtype, which is associated with a better clinical outcome (**Supplementary Figure S2D**). Notably, among the 47 markers, the serine peptidase inhibitor Kazal type I (*SPINK1*) showed enhanced expression in luminal UTUC but not in basal tumors (**Supplementary Figure S2E**). Immunohistochemistry showed higher expression of SPINK1, a potential luminal subtype-specific biomarker for UTUC (**Figure 2B**). Using the 47 genes, we defined two computational scores, the basal score and the luminal score, to quantify the two subtypes. In the Japanese UTUC cohort, the basal score was significantly associated with decreased disease-specific survival (DSS), whereas the luminal score was significantly associated with increased DSS in the univariate analysis (**Figure 2C**). Furthermore, the basal score and luminal score can generate “poor” or “good” prognosis calls for MI UCB in The Cancer Genome Atlas (TCGA) (**Supplementary Figure S2F**).

To further understand the gene expression patterns that differentiate the intrinsic subtypes of MI UTUC, we identified 521 differentially expressed genes (DEGs) in the tumor cells of these two subtypes (**Figure 2D, 2E & Supplementary Table S3**). Gene Ontology (GO) analysis showed that the upregulated genes in the luminal subtype were enriched for pathways related to the epithelium development. The upregulated genes in the basal subtype were enriched in immune regulation pathways, such as the response to interferon beta and the response to cytokine (**Figure 2F**). Interestingly, we observed enrichment of genes involved in regulating myeloid cell differentiation with upregulated expression of the myeloid chemotaxis signature, and immune evasion genes in the basal subtype compared to the luminal subtype (**Figure 2F & 2G**). We found that the basal subtype had a stronger immune evasion phenotype than the luminal subtype.

### An immunosuppressive macrophage population is enriched in the basal subtype and correlates with poor clinical outcome

**Figure 3:**
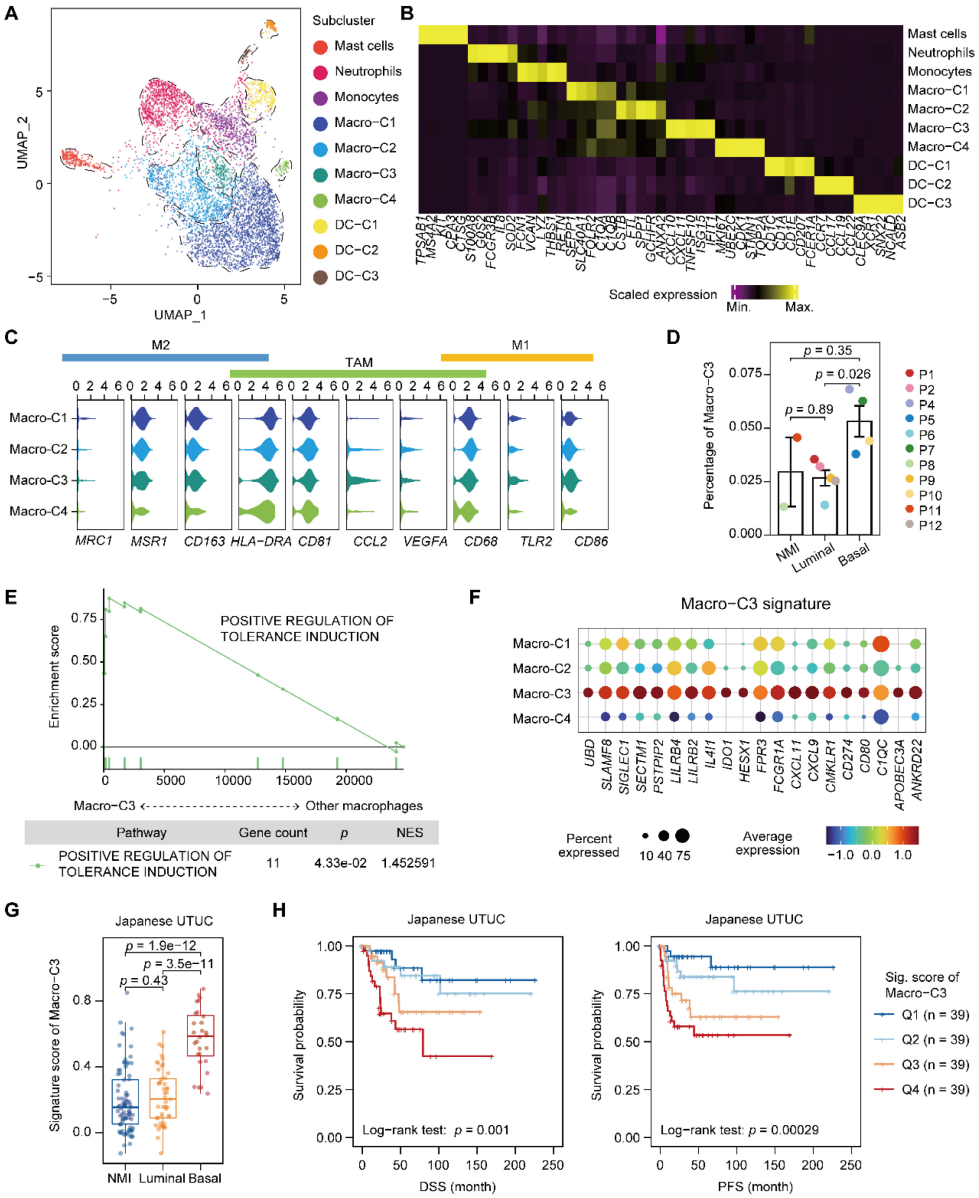
** Accumulation of an immunosuppressive macrophage population in the basal subtype. (A)** UMAP projections of 6,292 subclustered myeloid cells. **(B)** Heatmap of the scaled normalized expression of subcluster-defining genes as determined by the “MAST” method. **(C)** Violin plots showing the expression of M1, M2, and TAM markers in the macrophage populations. **(D)** Relative percentage of Macro-C3 cells (immunosuppressive macrophages) among the NMI, luminal, and basal subtypes. P3 was excluded due to the presence of <100 myeloid cells. The relative percentage of Macro-C3 cells is defined as the proportion of Macro-C3 in myeloid populations. Error bars represent the mean ± standard error of the mean. *P* values were calculated with a *t* test. **(E)** GSEA revealed the enrichment of the GO BP term positive response to tolerance induction in the Macro-C3 population compared with the other macrophage populations. NES, normalized enrichment score. **(F)** Dot plot of genes defining the Macro-C3 signature in macrophage subclusters. Colors indicate the scaled mean expression of a gene. The size of the circles indicates the percentage of cells expressing the gene. **(G)** The Macro-C3 signature score for the NMI, luminal, and basal subtypes in the Japanese UTUC cohort. *P* values were calculated with a *t* test. **(H)** Kaplan-Meier plot of the relationship of patient DSS and PFS with the Macro-C3 signature score for the Japanese UTUC dataset. Patients were stratified by score quartile, in which Q1 and Q4 had the lowest and highest scores, respectively. *P* values were calculated using the log-rank test.

We next characterized myeloid cells in UTUC since the tumor cells of the basal and luminal subtypes showed a discrepancy in the regulation of myeloid differentiation and migration (**Figure 3A, 3B, Supplementary Figure S3A-C, and Table S4**). Macrophages were the most dominant myeloid cell type in UTUC and expressed TAM markers, whereas subcluster-specific expression of conventional proinflammatory (M1) and anti-inflammatory (M2) macrophage markers were not observed (**Figure 3C**). Interestingly, we identified a subcluster of macrophages (Macro-C3) that was significantly enriched in the basal subtype (**Figure 3D**). This subcluster highly expressed CD8^+^ T-cell recruitment-related genes, such as *CXCL10* and *CXCL11,* as well as immune checkpoint and evasion genes (**Figure 3B & Supplementary Figure S3A**). Compared with other macrophages, Macro-C3 showed the upregulation of genes that could positively affect the induction of immune tolerance, indicating a central role in the ecosystem of UTUC (**Figure 3E**). This subcluster resembled recently identified immunosuppressive macrophages that consisted predominantly of genes associated with immunosuppression 18.

After identifying a subset of subtype-relevant macrophages, we explored their clinical implications. For this, a Macro-C3 signature was developed and consisted predominantly of genes associated with immunosuppression, such as *IDO1* and *CD274* (**Figure 3F, Supplementary Table S2 & S4**). Consistent with the observations in our scRNA-seq dataset, evaluation of the signature in the Japanese UTUC cohort revealed that the basal subtypes had significantly higher Macro-C3 signature scores than the NMI and luminal subtypes (**Figure 3G**). Additionally, higher Macro-C3 signature scores in the Japanese UTUC cohort were significantly correlated with reduced DSS and progression-free survival (PFS) (**Figure 3H**). Collectively, these findings suggest a shift toward enhanced immunosuppression of macrophages in basal UTUC.

### Expansion of exhausted T cells in the basal subtype

**Figure 4:**
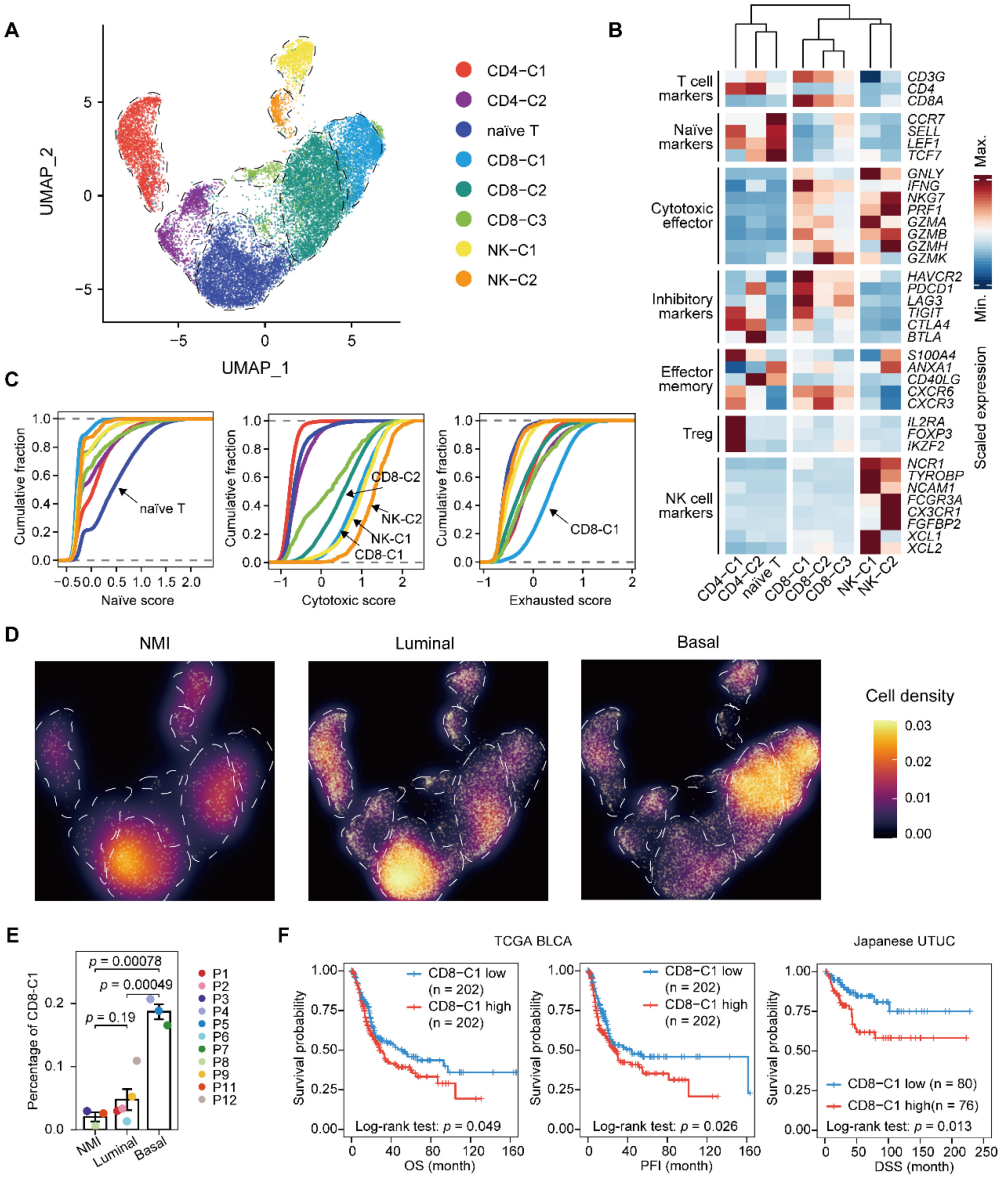
** Expansion of exhausted T cells in the basal subtype. (A)** UMAP projections of 20,862 subclustered T cells. **(B)** Heatmap indicating the expression of selected functionally relevant genes in the T/NK subtypes. **(C)** Cumulative distribution function showing the distribution of naïve, cytotoxic, and exhausted state scores in each T/NK subtype. A rightward shift of the curve indicates increased state scores. **(D)** Differences in the composition of the T/NK population among subtypes. Fractions are visualized as cell density based on the UMAP embedding. **(E)** Relative percentage of CD8-C1 cells (exhausted CD8^+^ T cells) among the NMI, luminal, and basal subtypes. P10 was excluded due to the presence of <100 T/NK cells. The relative percentage of CD8-C1 cells is defined as the proportion of CD8-C1 cells in the T/NK populations. Error bars represent mean ± standard error of the mean. *P* values were calculated with a *t* test. **(F)** Kaplan-Meier curves of OS and progression free interval (PFI) with the decomposed CD8-C1 (exhausted CD8^+^ T cells) proportions in the TCGA-BLCA cohort, and DSS in the Japanese UTUC cohort. Samples were categorized as high (red, top 50%) or low (blue, bottom 50%) CD8-C1 abundance. *P* values were calculated using the log-rank test.

Next, we performed unsupervised clustering of T and NK cells and obtained eight subclusters, including a population of naïve T cells, two clusters of CD4^+^ T cells, three clusters of CD8^+^ T cells, and two of NK cells **(Figure 4A-C & Supplementary Figure S4A-C)**. Evaluation of the cell scores revealed a subcluster of exhausted CD8^+^ T cells, CD8-C1, that was characterized by the expression of both inhibitory effectors and cytotoxic markers, indicating an activation-coupled exhaustion program 19 **(Figure 4B & 4C)**. In addition, there were CD8-C2 cells showing high *GZMK* expression and a small subset of CD8^+^ T cells, CD8-C3, that was marked by the expression of interferon-stimulated genes, including *ISG15* and *MX1*, and of transcripts encoding interferon-induced proteins with tetratricopeptide repeats (IFITs) **(Figure 4B & Supplementary Figure S4B)**. However, the CD4^+^ T cells were mainly regulatory (CD4-C1) or exhausted (CD4-C2) **(Figure 4B)**. To ascertain the developmental trajectory of the T cells, we performed a pseudotime analysis. The CD4^+^ T-cell branches originated from naïve T cells, which differentiated into regulatory T cells (CD4-C1) and exhausted CD4^+^ cells (CD4-C2) **(Supplementary Figure S4C)**. The CD8^+^ T cells, however, showed a different developmental trajectory, starting from the naïve state, passing through the cytotoxic phase (CD8-C2), and ending in exhaustion (CD8-C1) **(Supplementary Figure S4C & S4D)**.

Next, we sought to characterize the T lymphocytes in distinct UTUC subtypes. We found that the relative proportion of naïve T cells was significantly lower in the basal subtype than in the other two subtypes (**Figure 4D & Supplementary Figure S4E**). Moreover, the exhausted CD8^+^ T-cell population (CD8-C1) showed significant enrichment in the basal subtype, which indicated increased immune exhaustion and CD8^+^ T-cell dysfunction in the basal tumor microenvironment **(Figure 4E)**. A high proportion of exhausted CD8^+^ T cells was significantly associated with the poor survival of patients with UCB and UTUC according to single-cell deconvolution in the TCGA-BLCA and Japanese UTUC cohorts, respectively **(Figure 4F)**. Therefore, we concluded that CD8^+^ T cells in basal UTUC show the highest rate of exhaustion, suggesting weakened immune surveillance.

### The interaction between myeloid and lymphoid compartments shapes an immunosuppressive microenvironment in basal UTUC

**Figure 5:**
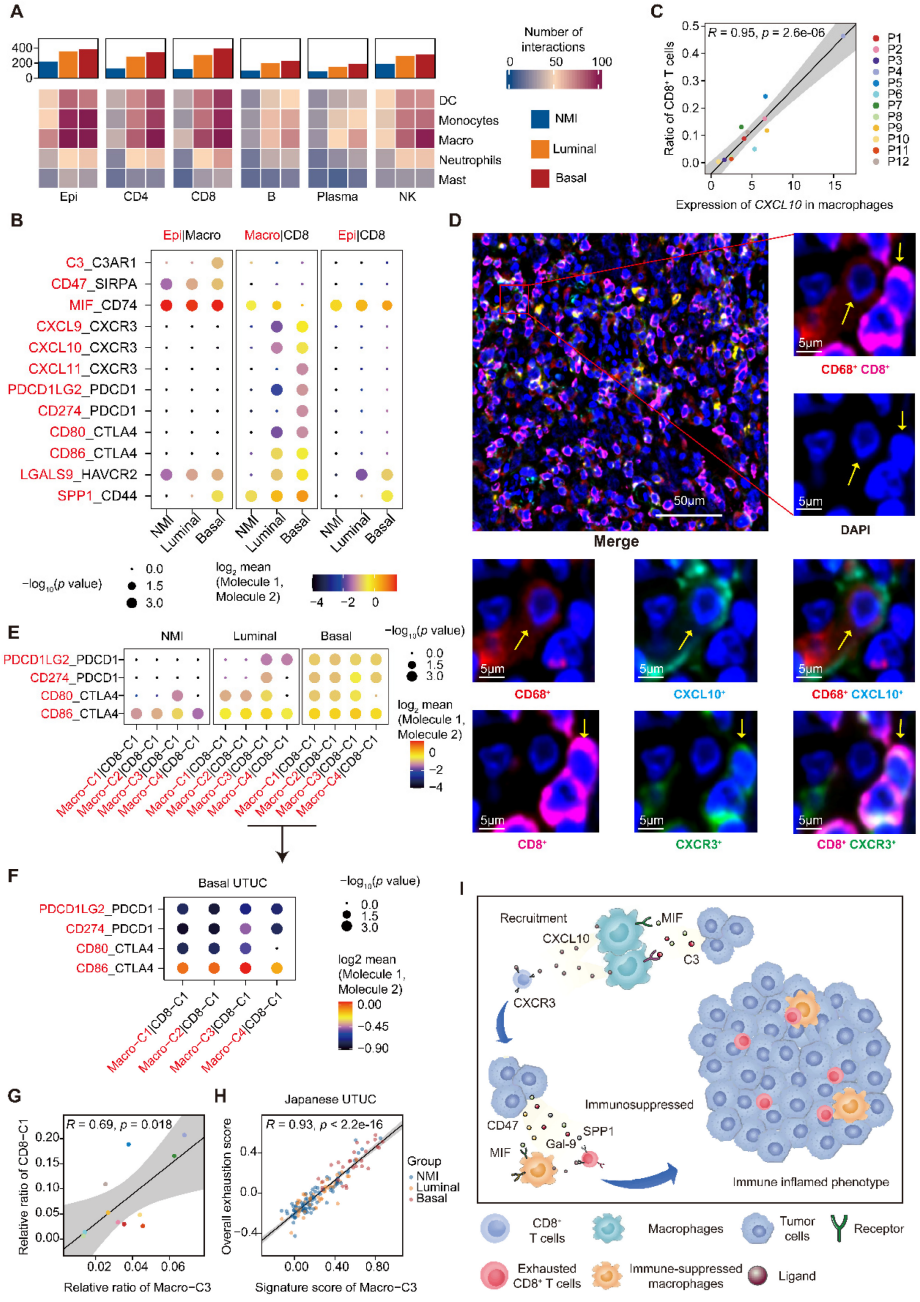
** Recruitment and exhaustion of CD8^+^ T cells by TAMs define the ecosystem of basal UTUC. (A)** Heatmaps showing the number of statistically significant ligand-receptor pairs between myeloid lineage cells and other cell types. The bar plots (top) summarize the overall number of interactions with myeloid cells for the indicated cell types in the UTUC subtypes. B/plasma cells were re-subclustered using a similar approach applied in T cell and myeloid cell subclustering. **(B)** Summary of selected ligand-receptor interactions between the tumor cells, CD8^+^ T cells, and macrophages. Circle size indicates the *P* value (permutation test). The color gradient represents the interaction strength. **(C)** Scatterplot showing the correlation between the expression of *CXCL10* in macrophages and the proportion of CD8^+^ T cells in the scRNA-seq dataset. **(D)** Representative immunofluorescence images illustrating the interaction between CD8^+^ T cells and macrophages in one UTUC sample (P4). The small panels show the magnification of the selected region highlighted in red. Yellow arrows indicate the colocalization of CD8^+^ T cells and macrophages. Scale bars correspond to 50 µm and 5 µm in the large and small panels, respectively. **(E and F)** Summary of selected ligand-receptor interactions between macrophages and CD8-C1 cells (exhausted CD8^+^ T cells) in the NMI, luminal (E), and basal subtypes (F). **(G)** Scatterplot showing the correlation between the relative ratio of Macro-C3 (immunosuppressive macrophages) and CD8-C1 cells (exhausted CD8^+^ T cells) in the scRNA-seq dataset. **(H)** Scatterplot showing the correlation between the signature score of Macro-C3 (immunosuppressive macrophages) and the overall exhaustion score in the Japanese UTUC cohort. Samples are colored according to subtype. **(I)** Schematic showing the crosstalk among CD8^+^ T cells, macrophages, and tumor-derived epithelial cells involved in the recruitment of immune cells and the formation of an immunosuppressive microenvironment in the basal subtype. In (C), (G), and (H), the Pearson coefficient (*R*) and associated *P* value are reported.

Given the common trends of immunosuppressive phenotypes in the malignant cells, macrophages, and T cells of the basal subtype compared to the luminal subtype, we hypothesized that different cell populations participate in complex crosstalk. Therefore, we firstly investigated possible intercellular interactions influencing TAMs. We found that macrophages had the most frequent communication with epithelial cells over other myeloid cells, especially in the basal subtype (**Figure 5A**). Between the two cell types, intense signaling mediated by *MIF* and *CD74* was identified, which has been reported to activate the recruitment of macrophages 20 and promote the secretion of growth factors by TAMs 21 (**Figure 5B**). Moreover, basal UTUC exhibited higher CD47-SIRPA and C3-C3AR1 interactions, which are known to inhibit phagocytosis 21 and associated with TAM infiltration 22, respectively (**Figure 5B**).

The active crosstalk between basal malignant cells and macrophages explained increased immunosuppression in this subtype, yet the process ruling T cell recruitment remains elusive. Therefore, we screened receptor-ligand pairs and found a significant positive correlation between CD8^+^ T-cell abundance, exhaustion and macrophage-derived *CXCL* expression (**Figure 5C & Supplementary Figure S5A-C**). Moreover, in the basal subtype we identified stronger macrophage-CD8^+^ T-cell interactions mediated by *CXCL* and their receptors (**Figure 5B**), which were essential for the recruitment of T cells into tumors 23. This association was further validated in the TCGA-BLCA and Japanese UTUC cohorts, where the basal subtype showed significantly enhanced *CXCL10* expression and a greater number of tumor-infiltrating CD8^+^ T cells and macrophages (**Supplementary Figure S5D**). Mechanistically, multiplex immunofluorescence staining revealed the colocalization of CD68-expressing macrophages (*CXCL10^+^*) and CD8-expressing CD8^+^ T cells (*CXCR3^+^*) in the basal subtype (**Figure 5D**) with a closer average distance compared to luminal UTUC (**Supplementary Figure S5E**), which further indicated that macrophages may play an important role in the recruitment of CD8^+^ T cells to UTUC.

Next, we explored the mechanism underlying the exhaustion of recruited CD8^+^ T cells. In the basal subtype, both tumor cells and macrophages showed enhanced LGALS9-HAVCR2, SPP1-CD44 interactions with CD8^+^ T cells (**Figure 5B**), which are known to suppress T-cell functions 24, 25. Furthermore, macrophages of the basal subtype expressed *PDCD1LG2*, *CD274*, *CD80*, and *CD86*, which target the immune checkpoints *CTLA-4* and *PDCD1* to inhibit CD8^+^ T-cell activation (**Figure 5B**). Interestingly, the exhausted CD8^+^ T cell population, CD8-C1, showed intensive signaling in the basal subtype with macrophages (**Figure 5E**), particularly Macro-C3 (**Figure 5F**). Consistently, the proportions of Macro-C3 and CD8-C1 were positively correlated (**Figure 5G**), implying a pivotal role of immunosuppressive macrophages in driving the exhaustion of CD8^+^ T cells. This association was further validated by analyzing the Japanese UTUC cohort, in which exhaustion scores and Macro-C3 signature scores also showed a strong correlation (**Figure 5H**). Collectively, this evidence reveals intense interactions between the myeloid and lymphoid compartments in UTUC and suggests that epithelial cells, macrophages, and T lymphocytes cooperate to shape the immunosuppressive microenvironment in the basal subtype with high infiltration of exhausted T lymphocytes (**Figure 5I**).

### Macro-C3 score predicts the immunotherapy response in mUC

**Figure 6:**
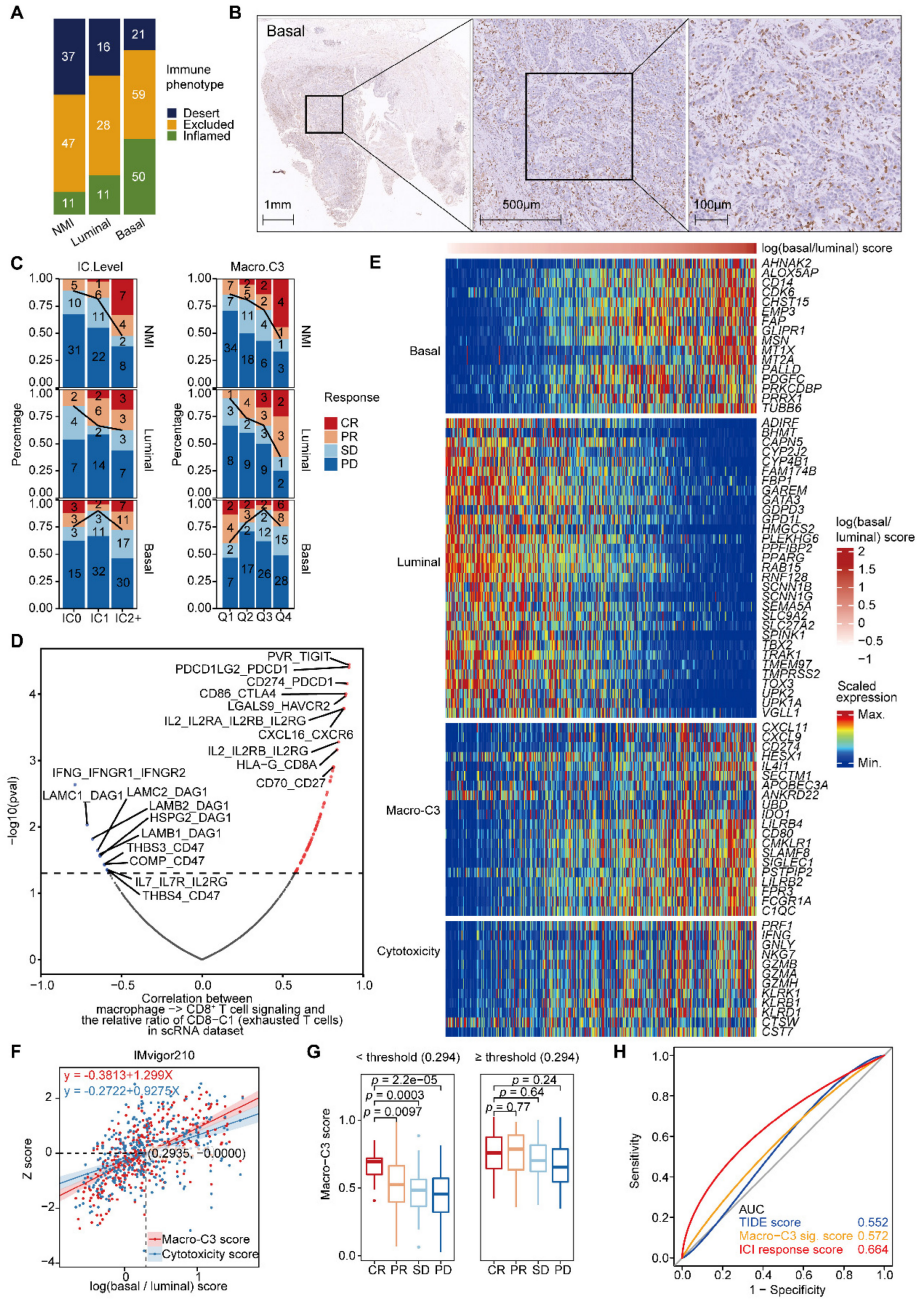
** The Macro-C3 score predicts the immunotherapy response in mUC. (A)** Bar plots showing the classification of subtypes associated with the immune phenotype in the IMvigor210 cohort. Sample sizes are given in the figure. **(B)** Representative CD8 staining shows the infiltration of CD8^+^ T cells (brown) in basal (P5) samples. Scale bars correspond to 1 mm, 500 µm, and 100 µm in the large and small panels, respectively. **(C)** PD-L1 on immune cells is associated with response in the NMI subtype (two-sided Fisher's exact test, NMI: *p* = 0.0009046), while the Macro-C3 score is associated with response in the NMI and luminal subtypes (Q1 versus Q4, NMI: *p* = 0.01466, luminal: *p* = 0.01806, basal: *p* = 0.3308) in the IMvigor210 cohort. Sample sizes are given in the figure. Patients were stratified by IC level or score quartile, wherein IC level stands for PD-L1 expression on tumor-infiltrating immune cells assessed by SP142 immunohistochemistry assay and scored as level 0 (< 1%), level 1 (≥ 1% and < 5%), or level 2+ (≥ 5%). Q1 and Q4 had the lowest and highest Macro-C3 scores, respectively. **(D)** Scatterplot showing the correlation of the interactions between macrophages and CD8^+^ T cells with the percentage of CD8-C1 cells (exhausted CD8^+^ T cells) in T/NK populations. The statistically significant interactions are highlighted in red and blue indicating positive and negative correlations, respectively. Top 10 positive or negative interactions are labeled. **(E)** Heatmap indicating the scaled expression of selected genes from different categories in the IMvigor210 cohort. The samples were ordered by log(basal/luminal) score. **(F)** Linear regression of the z score of overall cytotoxicity or Macro-C3 score on log(basal/luminal) score. The regression equations and the intersection point produced by the two regression lines are indicated in the figure. **(G)** Boxplot showing Macro-C3 signature score was significantly associated with response to ICIs for patients with log(basal/luminal) scores below the threshold (0.294) in the IMvigor 210 cohort. **(H)** ROC curves assessing the performance of the TIDE, Macro-C3 signature and ICI response scores in predicting the ICI response in the IMvigor 210 cohort. In (G), the center line in the boxplots indicates the median, the lower and upper hinges correspond to the first and third quartiles, and the whiskers extend at most 1.5 times the interquartile range past the upper and lower quartiles. The P value indicates a two-sided two group t test without adjustment.

To further evaluate the determinants of response and resistance to immunotherapy, we examined whether subtype-specific immunological phenotypes can predict the treatment response. Pretreatment tumor samples from a large phase II trial (IMvigor210) investigating the clinical activity of PD-L1 blockade with atezolizumab in metastatic urothelial cancer (mUC) were used for this integrated evaluation. We categorized patient tumors into the NMI, luminal, and basal subtypes. In the IMvigor210 dataset, a larger proportion of basal tumors (38%) than luminal (20%) or NMI (11%) tumors exhibited an inflamed phenotype (**Figure 6A**). Immunohistochemical staining further confirmed the inflamed phenotype in the basal subtype and the excluded phenotype in the luminal subtype (**Figure 6B & Supplementary Figure S6A**). PD-L1 expression on immune cells (more than 5% of immune cells in mUC were detected by the PD-L1 antibody SP142) and the Macro-C3 score were associated with the response in the NMI and luminal subtypes but not in the basal subtype (**Figure 6C**). A nonlinear relationship between the log(basal/luminal) score and fraction of responders to ICI treatment was identified (**Supplementary Figure S6B**). Thus, we hypothesized that immunosuppressive interactions between macrophages and T cells may contribute to immune evasion in the basal subtype.

Next, we examined the most significant interactions between macrophages and CD8^+^ T cells, which were positively and negatively associated with the abundance of exhausted CD8^+^ T cells. We found multiple known interactions, such as CD274-PDCD1, CD86-CTLA4, and LGALS9-HAVCR2, among the positively associated interactions that promoted T-cell exhaustion 26 (**Figure 6D & Supplementary Figure S6C**). Consistently, we identified multiple known interactions, such as IFNG-IFNGR1 and IL-7-IL7R 27, among the negatively associated interactions that reversed T-cell exhaustion (**Figure 6D**).

Moreover, we found that the expression of immunosuppressive Macro-C3 signature genes and T-cell cytotoxicity genes increased with an enhanced basal signature (**Figure 6E & Supplementary Figure S6D**). Therefore, we developed a computational method to model the extent of immune evasion using the Macro-C3 score and cytotoxic T cells using the T-cell cytotoxicity score (**Figure 6F**). We identified an intersection point between the z score of T-cell cytotoxicity and the Macro-C3 score (**Figure 6F**); based on the log(basal/luminal) value of this intersection, we divided the IMvigor210 cohort into two groups. Below this threshold, the Macro-C3 score and the fraction of responders to PD-L1 blockade treatment were significantly correlated; thus, we denoted the Macro-C3 signature as the ICI response score (**Figure 6G**). Above this threshold, the Macro-C3 score represented immune evasion capacity, and thus, we assigned the minimal Macro-C3 score of the cohort as their ICI response score. To evaluate the predictive performance regarding the ICI response, we compared the ICI response score with the Macro-C3 score and a published signature for predicting the clinical response to immune checkpoint blockade, termed the tumor immune dysfunction and exclusion (TIDE) score 28. The receiver operating characteristic (ROC) curves showed that the ICI response score had a higher AUC (0.664) than the TIDE score (0.552) (**Figure 6H**). Overall, the Macro-C3 score predicted the ICI response, especially in the NMI and luminal subtypes, whereas the Macro-C3 score predicted ICI resistance in the basal subtype, likely based on the enhanced T-cell dysfunction induced by immunosuppressive macrophages.

## Discussion

Herein, to the best of our knowledge, we have performed the first comprehensive profiling of UTUC at the single-cell level, which allowed us to simultaneously elucidate the cell state of malignant and nonmalignant cells and to derive more therapeutically relevant molecular signatures with higher resolution. Although UTUC is more frequently diagnosed in the locally advanced stage than UCB, we surprisingly found that the intrinsic subtype of tumor cells and subtype-specific immune contexture were similar in UTUC and UCB. Thus, it is reasonable to hypothesize that there are similar epithelial and/or nonepithelial subpopulations with the ability to predict the therapeutic response in both UTUC and UCB. Consistently, in a single-arm phase II study of atezolizumab in mUC (IMvigor210) 15, we identified a similar prediction signature for anti-PD-L1 therapy in the NMI, basal, and luminal subtypes.

It has been shown that among UTUC and UCB patients who receive atezolizumab (anti-PD-L1 therapy), the response rate is higher in UTUC than in UCB (39% vs. 17%) 29, 30. However, they found no significant differences in baseline covariates, including anatomic sites of metastases, tumor mutation load, T-effector gene expression, TCGA subtype, and baseline tumor burden, between the UTUC and UCB 29. The higher response rate in UTUC may be at least partially explained by its predominant luminal subtype with a lower inflamed immunosuppressive contexture than the basal subtype 31 (**Figure 2G, 5E & Supplementary Figure S7**). Additionally, anatomic differences, but not intrinsic subtype or immune contexture, may account for much of this disparity in muscle-invasive UTUC and UCB at diagnosis because the thinner smooth muscle covering of the upper tract may allow for more rapid progression to non-organ-confined disease.

Although the application of bulk gene expression signatures has shown promise in identifying subgroups of patients responsive to immunotherapy 16, yet they provided a limited mechanistic understanding of the cell types responsible for mediating clinical benefit. In contrast, at the single-cell resolution, we found progressive T-cell dysfunction and/or exhaustion, likely through enhanced interactions between immunosuppressive macrophages and T-cell subpopulations. We identified a macrophage population, Macro-C3, that strongly expressed several immunosuppressive effectors, showed the strongest putative interaction with exhausted CD8^+^ T cells and defined poor patient prognosis in UTUC.

More importantly, we identified diverse cell-cell interactions between T cells and macrophages that promoted or reversed T-cell exhaustion (**Figure 6D**). Thus, strategies to inhibit TAM functions, especially those of immunosuppressive Macro-C3, in combination with immunotherapy may have great potential for the treatment of UC patients. Indeed, both preclinical and clinical strategies targeting the tumor-promoting functions of TAMs in cancer are being developed 32. It has been reported that M2-like macrophages impede ICIs that were based on the PD-1/PD-L1 axis 33. Consistently, *PD-L1* was highly expressed in Macro-C3 cells. Meanwhile, other genes, such as *CXCL9*, *CXCL11*, and *IDO1*, derived from the Macro-C3 signature may also serve as therapeutic targets to ameliorate T-cell exhaustion. Then, we developed a computational method based on the Macro-C3 score to model the two primary mechanisms of tumor immune evasion and T-cell cytotoxicity. Of note, compared to the widely used ICI response markers, PD-L1 levels and the TIDE signature, the Macro-C3 score achieved better performance in the mUC cohort. One explanation for the better performance of the Macro-C3 score is that this score utilized cancer type-specific T-cell dysfunction regulators. A central limitation of this study was the relatively small cohort of patients employed to generate these signatures, and larger cohorts with scRNA-seq data of UC patients would be required. Although the prognostic and predictive values of the identified signatures were validated in multiple external cohorts, further studies to deconvolute other less-characterized immune cell types are necessary for identifying novel therapeutic targets that address immune dysfunction in UC.

## Conclusions

Taken together, our results show the common underlying transcriptional subtypes and immune microenvironments in UTUC and UCB, identifying potential opportunities for common management strategies, especially in terms of ICI for UC. Overall, our findings contribute to the understanding of the pathophysiology of UC and provide novel prognostic assessment strategies and individualized treatment recommendations for different UC subtypes.

## Methods

### Patients and samples

Tissue samples were obtained from the UTUC patients who received ureterectomy or radical nephroureterectomy at the Peking University First Hospital. Twelve primary UTUC tumor tissues (three low-stage and nine high-stage), along with one adjacent tumor thrombus, were included in this cohort (**Supplementary Table S1**). This study was approved by the Ethics Committee of Peking University First Hospital (Grant No. 2018[186]).

### Single-cell suspension preparation and droplet-based single-cell RNA sequencing

Tissue samples were processed immediately after the surgical resection. Single-cell suspensions were obtained by mechanical and enzymatic dissociation. By following the manufacturer's protocol, we used the Single Cell 3' Library and Gel Bead Kit V3.1 (10x Genomics, 1000075) and Chromium Single Cell B Chip Kit (10x Genomics, 1000074) to prepare barcoded scRNA-seq. Paired-end 150 bp reads were then generated by sequencing the libraries on the Illumina NovaSeq6000 platform (performed by CapitalBio Technology, Beijing).

### Quality control of the scRNA-seq data

CellRanger (version 4.0.0) was used to generate raw gene expression matrixes from the sequencing data of each sample based on the human reference version GRCh37. The R package Seurat (version 4.0.0) was used for downstream quality control 34. Only genes expressed at >3 cells and cells with >200 genes detected were kept in the expression matrix. We then removed low-quality cells that met at least one of the following criteria: (i) >5000 expressed genes, (ii) <200 expressed genes, or (iii) >25% UMIs derived from the mitochondrial genome.

### Integration of multiple scRNA-seq datasets

The R package Seurat (version 4.0.0) was used for the dataset integration. Briefly, the function NormalizeData was applied to each expression matrix for log-transformation and the function FindVariableFeatures was used to select the top 2000 genes with high cell-to-cell variation. Then, “anchors” between individual datasets were identified using the function FindIntegrationAnchors. Based on these anchors, an unbatched dataset was created using the function IntegrateData.

### Unsupervised clustering and dimension reduction

We used the function ScaleData to scale and center the expression of 2000 variable genes, on which principal component analysis (PCA) was subsequently performed. Then, functions FindNeighbors and FindClusters were used for the first-round cluster on the first 20-50 PCs with a resolution of 0.1-0.6. Finally, the main cell clusters were identified with a resolution of 0.3, and nonlinear dimensional reduction was visualized using the two-dimensional uniform manifold approximation and projection (UMAP).

### Cell type annotation and cluster marker identification

The major cell types were first annotated using the SingleR (version 1.4.1) R package based on the expression of canonical cell type markers 35. Preferentially expressed genes in each cluster were identified using the FindAllMarkers function and differential expression testing was performed with the “MAST” method with sample identity as a latent variable 36. Cells that expressed two canonical cell type markers were classified as doublet cells, which were excluded from further analyses.

### CNV estimation of the malignant cells

CD45^-^ epithelial cells were analyzed using the inferCNV R package (version 1.11.1) to estimate copy number aberrations in scRNA-seq data 37. The EPCAM^-^ immune and stromal cells were used as references. A cut-off of 0.1 was used for the min average read counts per gene among reference cells. The option “random_trees” was used to define the tumor sub-clusters.

### Pathway analysis

Gene set variation analysis (GSVA) was performed using the GSVA R package (version 1.38.2) to estimate the enrichment scores of biological pathways 38. The hallmark and gene ontology (GO) gene sets were obtained using the R package msigdbr (version 7.4.1) and from the MsigDB website (http://software.broadinstitute.org/gsea/msigdb) 39. The limma R package was then used to evaluate the differential activities of pathways between different clusters or groups (*P* value < 0.05) 40.

DEGs between the two subtypes of cells were identified using the FindMarkers function of the Seurat R package with the same parameters used for FindAllMarkers. When analyzing the malignant epithelial cells of different subtypes, we overcame the intertumor heterogeneity by keeping only high-confidence DEGs for a subtype such that the expression in each sample had a >0.8 log_2_ fold change over the average expression of the other subtype. Overrepresentation analysis of gene ontology (GO) biological process (BP) terms was performed using the fora function of the fgsea R package (version 1.16.0) 41.

### Diffusion map and pseudotime analysis

PCA embeddings were extracted from the integration produced by Seurat R package. The destiny R package (version 3.1.1) was then used to produce the diffusion trajectory from the predefined PCA embeddings for pseudotime analysis. Naïve T cells were chosen as the root, and the function DPT was used to estimate the diffusion pseudotime (DPT) for single cells.

### Cell-cell communication analysis

We used CellphoneDB (version 2.0.0) 42 to explore the potential communication between different cell types based on the expression of ligand-receptor pairs. The receptor or ligand should be expressed by more than 10% of cells in a cluster to be included in the downstream analysis. To assess the significance of a ligand-receptor pair between two clusters, an empirical *P* value was determined by randomly assigning the cluster labels of each cell for 1,000 times. The R package CellChat (version 1.1.3) 43 was used to characterize the interaction number and strength with default parameters.

### Prediction of ICI response

The putative ICI response of patients in the mUC cohort was predicted based on the TIDE as well as the in-house developed Macro-C3 gene signature. For the TIDE evaluation, gene expression in the TPM was supplied and force-normalized. The cancer type was set to “other”.

To predict the response to ICI based on the Macro-C3 gene signature, enrichment scores were first estimated based on log-normalized TPM values using ssGSEA. For a cohort of size *N* comprising a group of *R* responders, the luminal, basal, cytotoxicity, and Macro-C3 gene signature scores were *L* = {*l*_1_, *l*_2_, …, *l_N_*}, *B* = {*b*_1_, *b*_2_, …, *b_N_*}, *C* = {*c*_1_, *c*_2_, …, *c_N_*}, and *M* = {*m*_1_, *m*_2_, …, *m_N_*}. The z-score of the cytotoxicity score is:



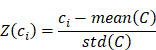



We then fitted a regression line *L_C_* taking the log(basal/luminal) score as the independent variable and *Z*(*C*) as the response. The same procedure was repeated for *Z*(*M*), which produced another regression line, *L_M_* intersecting *L_C_* at (*a*, *b*). This allows us to define an optimum log(basal/luminal) threshold *a* such that patients below this threshold have a relatively higher *Z*(*C*) compared to *Z*(*M*) and vice versa. Subsequently, we denoted the Macro-C3 signature as the ICI response score for patients with a log(basal/luminal) score < *a*:



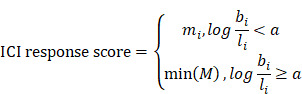



To prove that the log(basal/luminal) threshold *a* had a clinical implication, we generated 256 evenly distributed cutoff points of the log(basal/luminal) score *X* = {*x*_1_, *x*_2_, …, *x*_256_}. For a given cut-off point *x_j_*, we wrote the fraction of responders as follows:



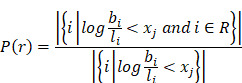



The log(basal/luminal) threshold *a* was then visualized to compare with *x_j_*.

The R package pROC (version 1.17.0.1) was used to generate ROC curves visualizing the true-positive rates versus false-positive rates at various thresholds of the TIDE, Macro-C3 signature, and ICI response scores. The area under the ROC curve (AUC) was adopted as the quality metric of prediction.

### Statistical analysis

Statistical analysis was performed using the R (version 4.0.3) and Python (version 3.8.8) and has been described in the Methods section and figure legends. Unless otherwise specified, the statistical analyses were performed in a two-sided manner. *P* < 0.05, *P* < 0.01, *P* < 0.001, and *P* < 0.0001 were considered statistically significant (*, **, ***, ****).

## Supplementary Material

Supplementary materials and methods, figures.

Supplementary tables.

## Abbreviations

UTUC: Upper tract urothelial carcinoma
UC: Urothelial carcinoma
UCB: UC of the bladder
mUC: metastatic UC
MI: muscle-invasive
NMI: non-muscle-invasive
TAMs: tumor-associated macrophages
PCA: principal component analysis
