# Supplementary material for: Single-cell atlases link macrophages and CD8+ T-cell subpopulations to disease progression and immunotherapy response in urothelial carcinoma
Source: Theranostics. 2022 Nov 14;12(18):7745–59. doi: 10.7150/thno.77281 (PMC9706581; doi:10.7150/thno.77281)
Supplement: Supplementary file 1 — Supplementary materials and methods, figures. [file thnov12p7745s1.pdf]

Supplementary Methods

Quality control of scRNA-seq data

Ambient RNA contamination was estimated and cleaned using the function autoEstCont and adjustCounts of the soupX R package (version 1.5.2) with default parameters [1]. Potential doublets in the corrected expression matrix were detected and removed using the scDblFinder R package (version 1.4.0) with default configuration [2].

Whole-genome sequencing

For whole-genome sequencing (WGS), genomic DNA was obtained from cancer tissue samples using the QIAmp DNA Mini Kit (QIAGEN Inc., MD, US). The sheared DNA was repaired and 3' dA-tailed using the NEBNext Ultra II End Repair/dA-Tailing Module unit and then ligated to paired-end (PE) adaptors using the NEBNext Ultra II Ligation Module unit. After purification by AMPure XP beads, the DNA fragments were amplified by PCR for 6-8 cycles. Finally, the libraries were sequenced with an Illumina HiSeq X10 instrument, thus generating 2 x 150-bp PE reads. The quality of short DNA reads was controlled by Trimmomatic. The good-quality PE reads were aligned with the human reference genome hg19 (http://genome.ucsc.edu/) using the BWA-MEM tool. The reads mapped with the reference at the same coordinates were removed using Picard.

CNV calling

Clean WGS data were used for CNV calling based on the varbin algorithm [3]. Briefly, the genome was divided into 50,000 equally mappable bins of variable lengths. Next, reads mapped to the genomic bins were counted. The counts were then normalized by the mean, corrected for GC content and segmented to produce CNV calls. R package ichorCNA (version 0.3.2) was used to validate the obtained CNV pattern [4]. The CNV profiles were visualized in the Integrative Genomics Viewer (IGV, version 2.8.13) [5].

Expression signature analysis

To evaluate the degree to which individual cells expressed a certain predefined expression gene set, we used the AddModuleScore function in Seurat with default settings to calculate the signature scores. The naïve, cytotoxicity, and exhaustion scores were measured based on well-defined naïve markers (*CCR7*, *TCF7*, *LEF1*, and *SELL*), cytotoxicity-associated genes (*PRF1*, *IFNG*, *GNLY*, *NKG7*, *GZMB*, *GZMA*, *GZMH*,

KLRK1, *KLRB1*, *KLRD1*, *CTSW*, and *CST7*), and exhausted markers (*LAG3*, *TIGIT*, *PDCD1*, *CTLA4*, *HAVCR2*, and *TOX*). For bulk RNA-seq data, the signature scores were evaluated using ssGSEA function of the GSVA R package.

Analysis of public RNA-seq datasets

Three RNA-seq datasets were included for external validation (**Supplementary Figure S7**): (i) the bladder urothelial carcinoma (BLCA) dataset from The Cancer Genome Atlas (TCGA, https://www.cancer.gov/tcga) [6], (ii) a Japanese cohort consisting of 158 individuals diagnosed with UTUC form the European Genome Phenome Archive under the accession number EGAD00001007667 [7], (iii) a metastatic UC cohort (IMvigor210) consisting of 348 patients received PD-L1 blockade with atezolizumab [8]. The TCGA expression data for BLCA were downloaded with TCGAbiolinks R package (version 2.18.0) [9] and curated clinical outcome endpoints were obtained from a previous study [10]. The IMvigor210CoreBiologies package was used to extract clinical information and gene expression profile for the IMvigor210 cohort.

To obtain a Macro-C3 gene expression signature for projection into bulk RNA-seq, we adopted a previously published selection method [11]. Briefly, genes significantly upregulated in Macro-C3 relative to other myeloid cells (with false discovery rate < 0.05, $\log2(\text{fold-change}) > 0.25$) were selected and restricted to myeloid-specific genes. Myeloid-specific genes were defined as the genes expressed in <5% of epithelial cells, lymphoid cells, endothelial cells, or fibroblasts.

To validate the profile of T lymphocyte and macrophage populations observed in our study, we performed single-cell deconvolution with public datasets based on Transcript Per Million (TPM) values. Briefly, the BASE47 gene set was used to classify basal and luminal subtypes in these datasets [12]. And the function ReferenceBasedDecomposition of the R package BisqueRNA (version 1.0.5) was employed to evaluate the relative abundance of each cell type in NMI, luminal and basal samples, respectively [13].

In survival analysis, samples with incomplete clinical data were removed. All events were considered regardless of radiological or pharmaceutical treatment received. For the Japanese UTUC cohort, a patient with bilateral tumors was excluded. Kaplan-Meier survival analyses were performed using the survival (version 3.2.7) and survminer (0.4.9) R packages where the estimated abundance was categorized into a "high" or "low" group using the median or quartiles as cutoffs. The univariate Cox proportional hazard regression was performed using the coxph function of the R package survival and the result was visualized

in forest plots using the R package forestmodel (version 0.6.2).

Immunohistochemistry and Immunofluorescence staining assays

Formalin-fixed, paraffin-embedded tissue samples, were cut into 5 μm sections and adhered on the glass slides heated at 70°C for 1h in Peking University First Hospital. Then, the paraffin-embedded slides were deparaffinized in xylene, and then rehydrated in 100%, 95%, 70% alcohol in sequence. Subsequently, the slides were processed further for either immunohistochemistry (IHC) or multiplex immunohistochemistry (mIHC) staining assays.

Immunohistochemistry staining assays were conducted to determine the distribution and expression of SPINK1 and CD8 using Rabbit Hypersensitive Two-step Detection Kit (ZSGB-Bio, China; Cat. no. PV-9001) and Mouse Hypersensitive Two-step Detection Kit (ZSGB-Bio, China; Cat. no. PV-9002), respectively, according to the manufacturer's instructions. In brief, the slides were subjected to the blockade of endogenous peroxidase activity for 10 min, and antigen retrieval of Citric acid solution with microwave for 15 min. After a pre-incubation with blocking buffer at room temperature for 10 min, the slides were incubated 1h at 37°C and 30 min at room temperature with primary antibodies, and then incubated with the secondary antibody (HRP polymer, anti-mouse/rabbit IgG) at room temperature for 20 min, followed by DAB colored, hematoxylin counterstained and microscopy. Primary antibodies included anti-SPINK1 (rabbit; Abcam; Cat. no. ab207302; 1:1000) and anti-CD8 (mouse; Proteintech; Cat. no. 66868-1-Ig; 1:5000).

To confirm the spatial contact of $CD8^+$ $CXCR3^+$ and $CD68^+$ $CXCL10^+$ cells, multiplex immunofluorescence staining was performed using Opal 7-color Manual IHC Kit (PerkinElmer; Cat. no. NEL811001KT) according to the manufacturer's instructions. In brief, the slides were subjected to the blockade of endogenous peroxidase activity for 10 min, and antigen retrieval of Citric acid solution with microwave for 20 min. After a pre-incubation with blocking buffer at room temperature for 10 min, the slides were incubated 1h at room temperature with primary antibodies (anti-CXCR3, anti-CD8, anti-CD68), but incubated overnight at 4°C with primary antibody (anti-CXCL10), and then incubated with the secondary antibody (HRP polymer, anti-mouse/rabbit IgG) at room temperature for 20 min. Subsequently, fluorophore (tyramide signal amplification or TSA plus working solution) was applied to the slides at room temperature for 10 min, followed by heat-treatment with microwave as before. The primary antibodies were applied

sequentially, followed by incubation with the secondary antibody and TSA treatment. Nuclei were stained with DAPI after all the antigens had been labelled. Multispectral images for each stained slide were captured using the Mantra System (PerkinElmer, USA). Primary antibodies included anti-CD8 (mouse; Proteintech; Cat. no. 66868-1-Ig; 1:5000), anti-CXCR3 (rabbit; Abcam; Cat. no. ab288437; 1:1000), anti-CD68 (rabbit; Abcam; Cat. no. ab213363; 1:5000), anti-CXCL10 (rabbit; Proteintech; Cat. no. 10937-1-AP; 1:500). HALO software (Indica Labs, https://indicalab.com/halo/) was used to plot representative immunofluorescence images. We randomly extracted 16 and 20 fields of view from the luminal (P9) and basal (P4) multiplex immunofluorescence images, respectively, with the luminal having a lower number of fields of view due to the smaller tumor area, and calculated the average distance between the two types of cells using HALO software.

Supplementary Figures

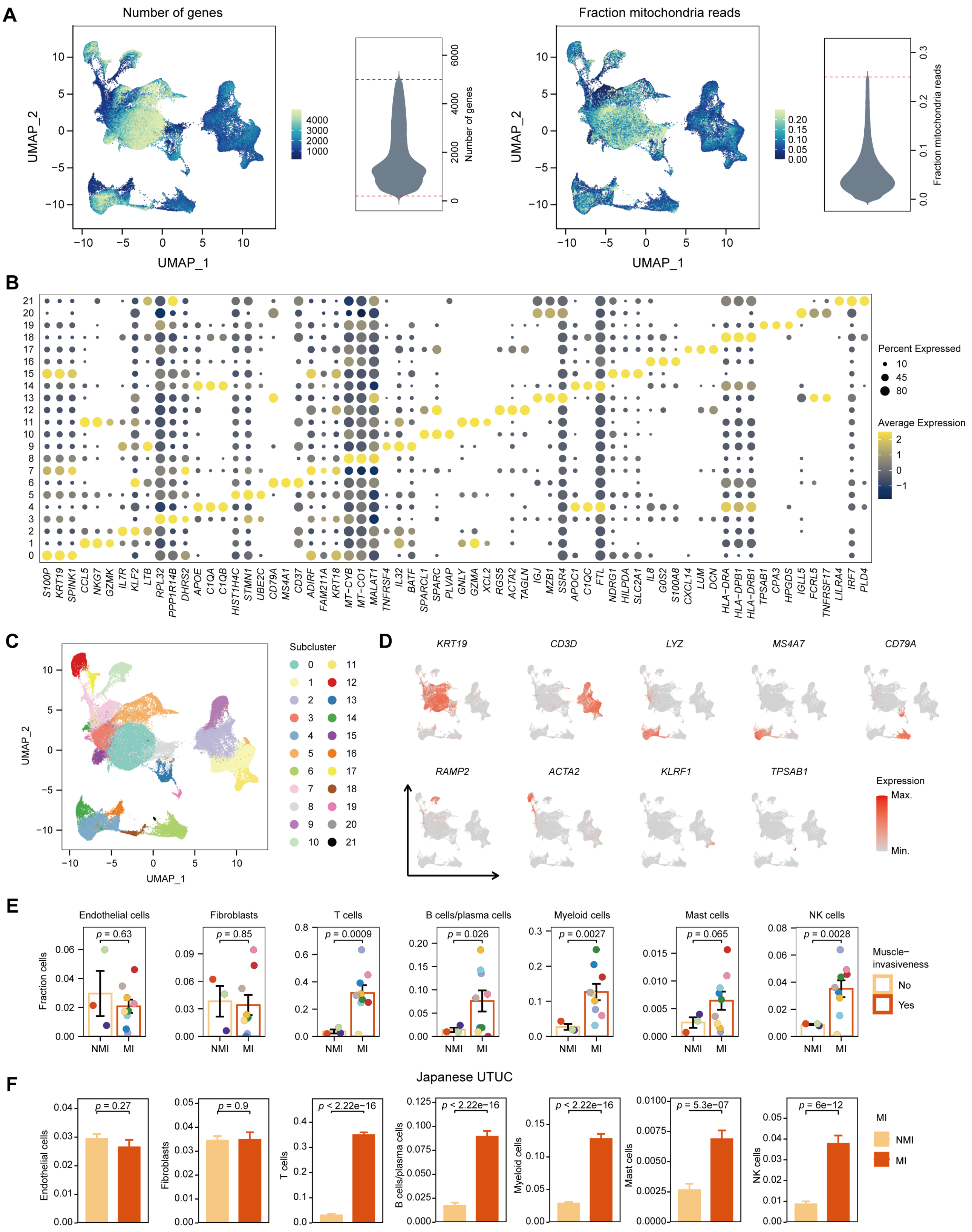

Figure S1. Identification of cell clusters and lineages in the scRNA-seq dataset. Related to Figure 1

(A) Violin plots and UMAP indicating number of genes and the percent mitochondria reads that were detected for 74,573 post-QC droplets.

(B) Dot plot showing the expression of the top 3 most specific marker genes for each cluster. Colors indicate scaled mean expression of a gene. Size of circles shows percentage of cells expressing the gene.

(C) UMAP of 22 sub-clusters.

(D) UMAP expression plot of canonical cell type markers used to identify major cell lineages.

(E) Percentages of major cell types for NMI and MI UTUC samples. The tumor thrombus (P4TT) was excluded in the analysis.

(F) Decomposed percentages of major cell types for NMI and MI samples in Japanese UTUC cohort.

In (E) and (F) error bars represent mean ± standard error of the mean. *P* values were produced with *t*-test.

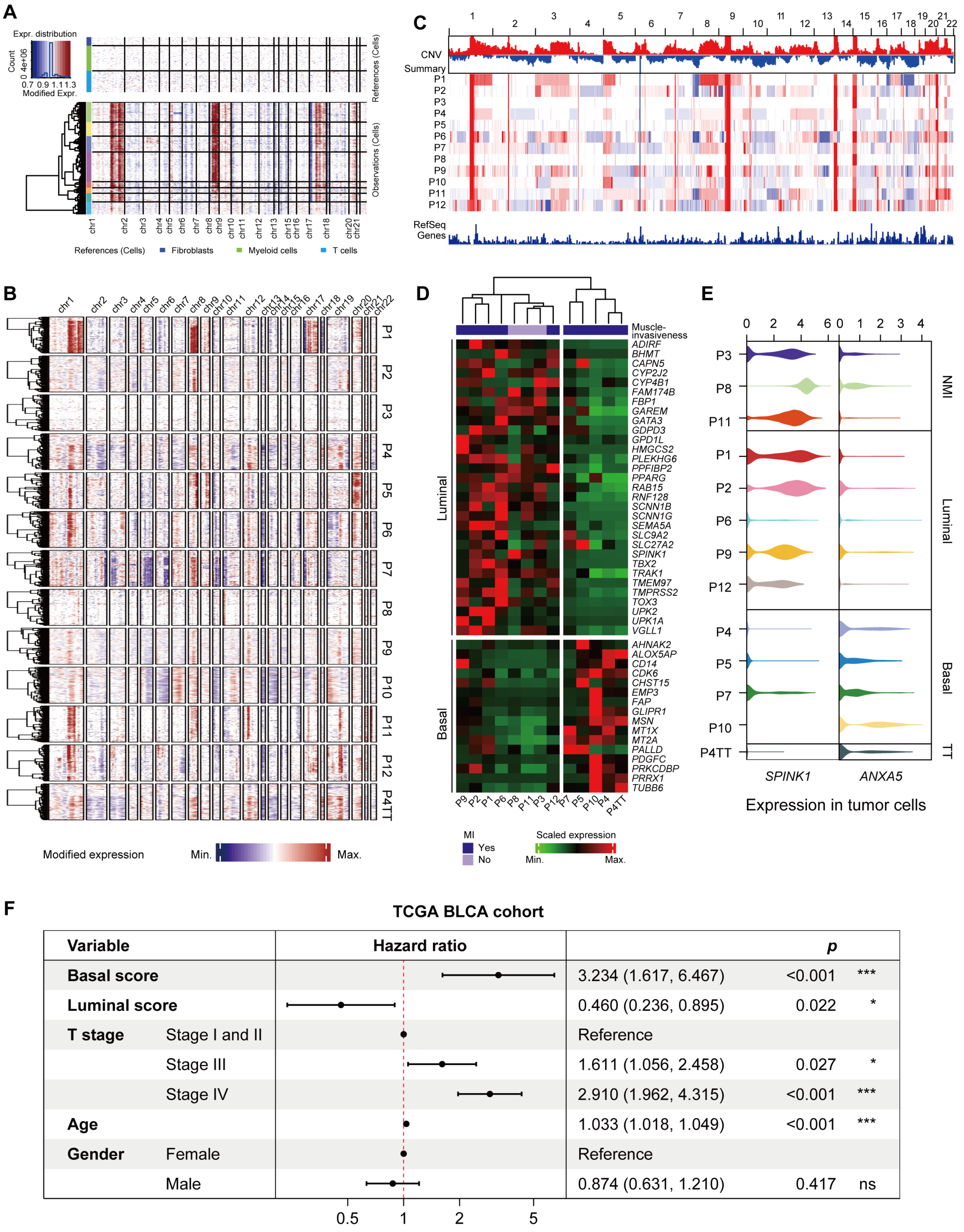

Figure S2. Characterization of tumor-derived epithelial cells. Related to Figure 2

(A) Heatmap showing inferred CNVs for individual epithelial cells (rows) from one UTUC sample (P1).

Immune cells and stromal cells were used as references (top). Colors represent the $\log_2$(CNV ratio). Red: amplifications; blue: deletions.

(B) Heatmap summarizing inferred CNVs for individual epithelial cells (rows) of UTUC samples. Colors represent the $\log_2$(CNV ratio). Red: amplifications; blue: deletions.

(C) WGS derived CNV shown in IGV. Chromosomes are labeled from 1 to 22 (top). Each row represents one sample. Colors represent the copy number. Red: amplifications; blue: deletions.

(D) Clustering analysis of UTUC samples based on the average expression levels of BASE47 genes in epithelial cells. The dendrogram tree is cut into two groups to reflect the classification efficacy of BASE47.

(E) Violin plots showing the expression of *ANXA5* and *SPINK1* in tumor-derived epithelial cells of UTUC samples.

(F) Forest plots showing hazard ratios associated with luminal score, basal score and clinical information in univariate Cox proportional hazard models for OS in the TCGA BLCA cohort.

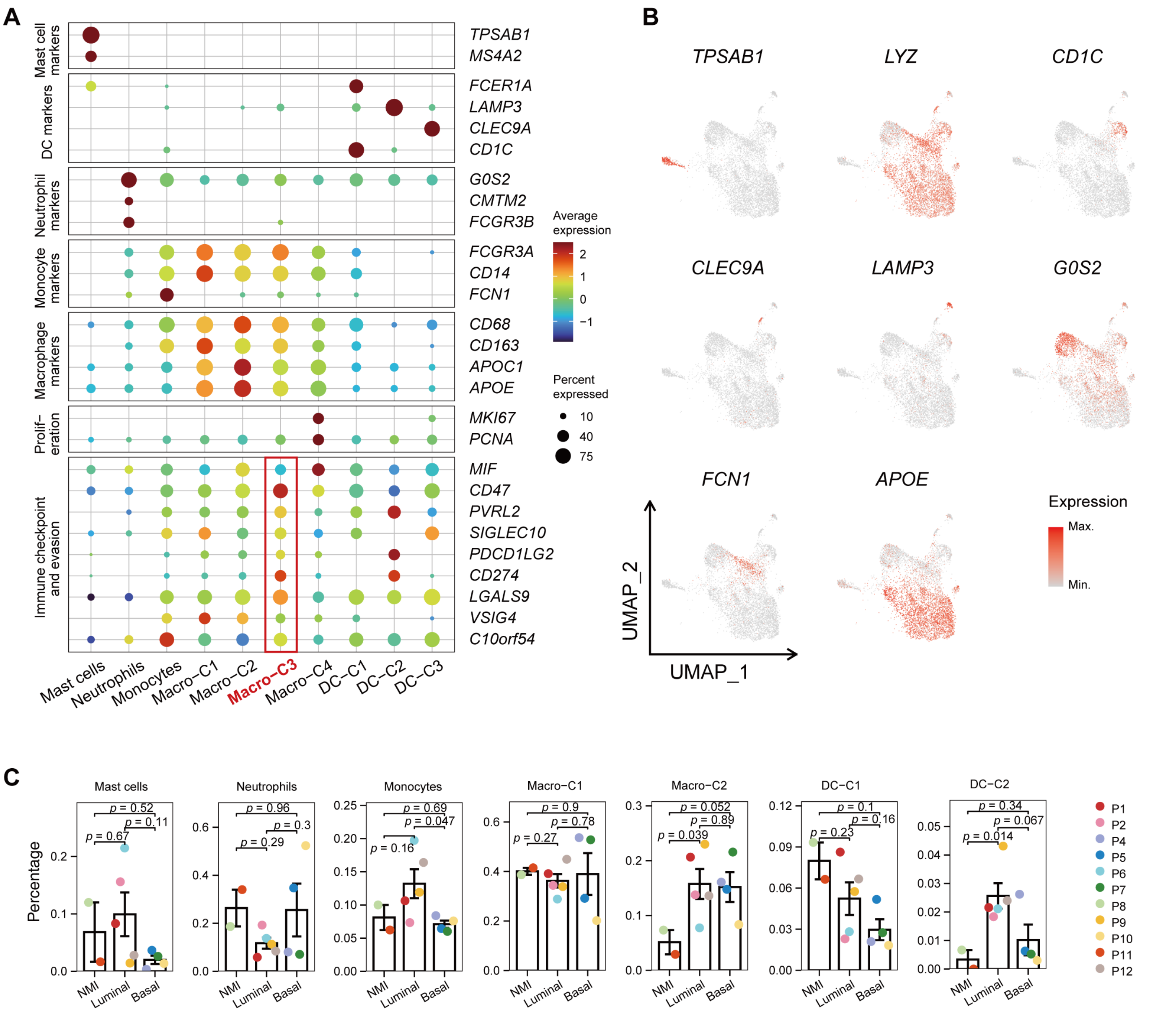

Figure S3. Myeloid cells in UTUC. Related to Figure 3

(A) Dot plot of selected gene sets in myeloid sub-clusters. Colors indicate scaled mean expression of a gene. Size of circles shows percentage of cells expressing the gene.

(B) UMAP expression plot of cell type markers used to identify myeloid cell subtypes.

(C) Relative percentages of myeloid subclusters among the NMI, luminal, and basal subtypes. P3 was excluded for <100 myeloid cells. Relative percentages are defined as proportions of subclusters in myeloid populations. Error bars represent mean ± standard error of the mean. *P* values were produced with *t*-test.

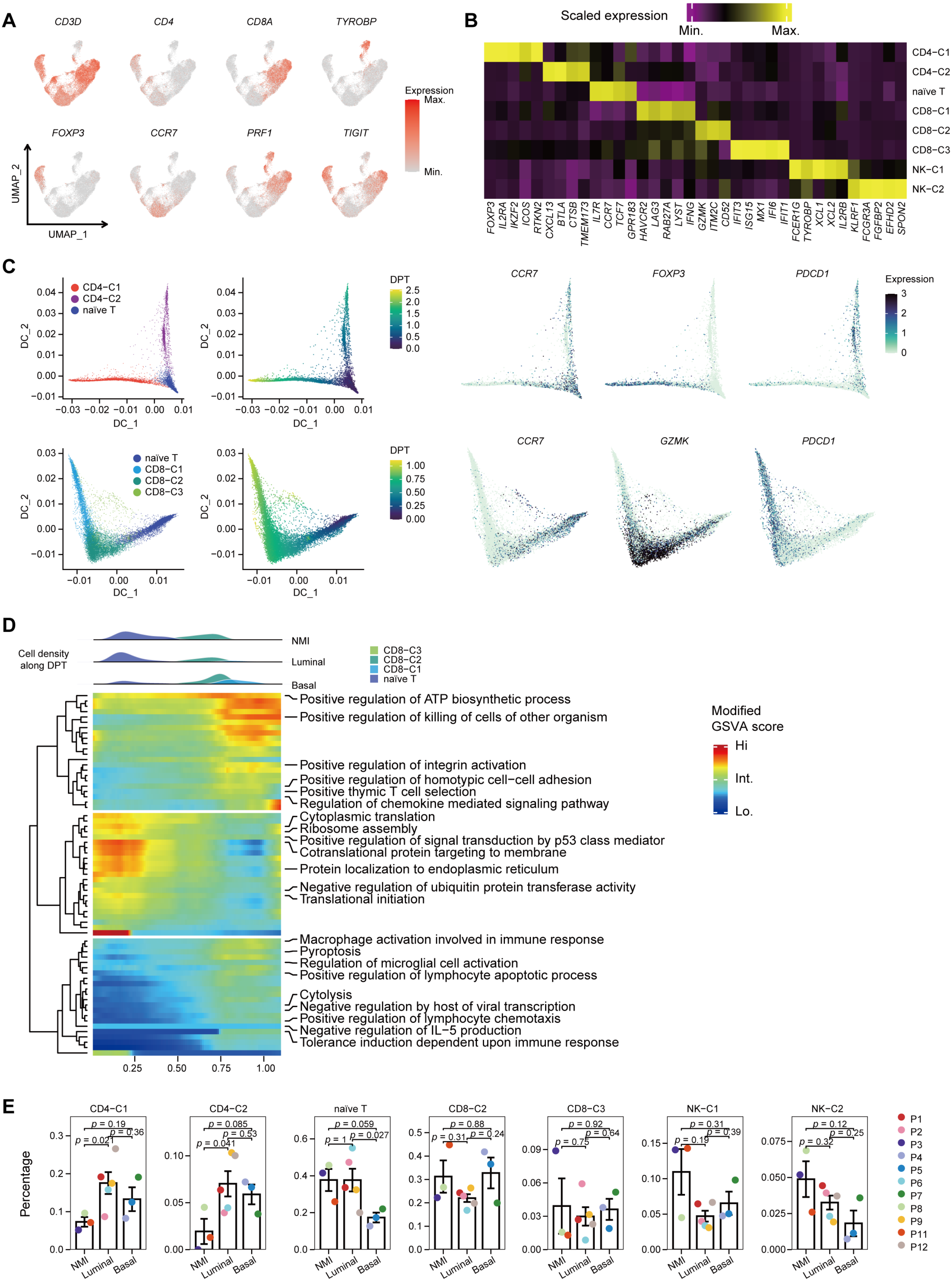
A
CD3D
CD4
CD8A
TYROBP
FOXP3
CCR7
PRF1
TIGIT
Expression
Max.
Min.
UMAP_2
UMAP_1
B
Scaled expression
Min.
Max.
CD4−C1
CD4−C2
naïve T
CD8−C1
CD8−C2
CD8−C3
NK−C1
NK−C2
FOXP3 IL2RA IKZF2 ICOS RTKN2 CXCL13 BTLA CTSB TMEM173 IL7R CCR7 TCF7 GPR183 HAVCR2 LAG3 RAB27A LYST IFNG GZMK ITM2C CD52 IFIT3 ISG15 MX1 IFI6 IFIT1 FCER1G TYROBP XCL1 XCL2 IL2RB KLRF1 FCGR3A FGFBP2 EFHD2 SPON2
C
CD4−C1
CD4−C2
naïve T
DC_2
DC_1
DPT
CCR7
FOXP3
PDCD1
Expression
naïve T
CD8−C1
CD8−C2
CD8−C3
CCR7
GZMK
PDCD1
D
Cell density along DPT
NMI
Luminal
Basal
CD8−C3
CD8−C2
CD8−C1
naïve T
Positive regulation of ATP biosynthetic process
Positive regulation of killing of cells of other organism
Positive regulation of integrin activation
Positive regulation of homotypic cell−cell adhesion
Positive thymic T cell selection
Regulation of chemokine mediated signaling pathway
Cytoplasmic translation
Ribosome assembly
Positive regulation of signal transduction by p53 class mediator
Cotranslational protein targeting to membrane
Protein localization to endoplasmic reticulum
Negative regulation of ubiquitin protein transferase activity
Translational initiation
Macrophage activation involved in immune response
Pyroptosis
Regulation of microglial cell activation
Positive regulation of lymphocyte apoptotic process
Cytolysis
Negative regulation by host of viral transcription
Positive regulation of lymphocyte chemotaxis
Negative regulation of IL−5 production
Tolerance induction dependent upon immune response
Modified GSVA score
Hi
Int.
Lo.
E
Percentage
CD4−C1
p = 0.19
p = 0.021
p = 0.36
CD4−C2
p = 0.085
p = 0.53
p = 0.041
naïve T
p = 0.059
p = 1
p = 0.027
CD8−C2
p = 0.88
p = 0.31
p = 0.24
CD8−C3
p = 0.92
p = 0.75
p = 0.64
NK−C1
p = 0.31
p = 0.19
p = 0.39
NK−C2
p = 0.12
p = 0.32
p = 0.25
NMI
Luminal
Basal
P1
P2
P3
P4
P5
P6
P7
P8
P9
P11
P12

Figure S4. T and NK cells in UTUC. Related to Figure 4

(A) UMAP expression plot of cell type markers used to identify T/NK subtypes.

(B) Heatmap of scaled normalized expression for subcluster-defining genes as determined by the "MAST" method.

(C) Development trajectory of $CD4^+$ and $CD8^+$ T cells constructed by diffusion map, colored by subcluster, diffusion pseudotime and the expression of cell-type-relevant genes.

(D) Heatmap displaying enrichment scores of selected GO BP terms in $CD8^+$ cells that are arranged along the pseudotime trajectory. Colors represent GSVA enrichment scores that were smoothed with locally-weighted polynomial regression and interpolated at evenly distributed diffusion pseudotime.

(E) Relative percentages of T/NK subclusters among the NMI, luminal, and basal subtypes. The tumor thrombus (P4TT) was excluded in the analysis. P10 was excluded for <100 T/NK cells. Relative percentages are defined as proportions of subclusters in T/NK populations. Error bars represent mean ± standard error of the mean. *P* values were produced with *t*-test.

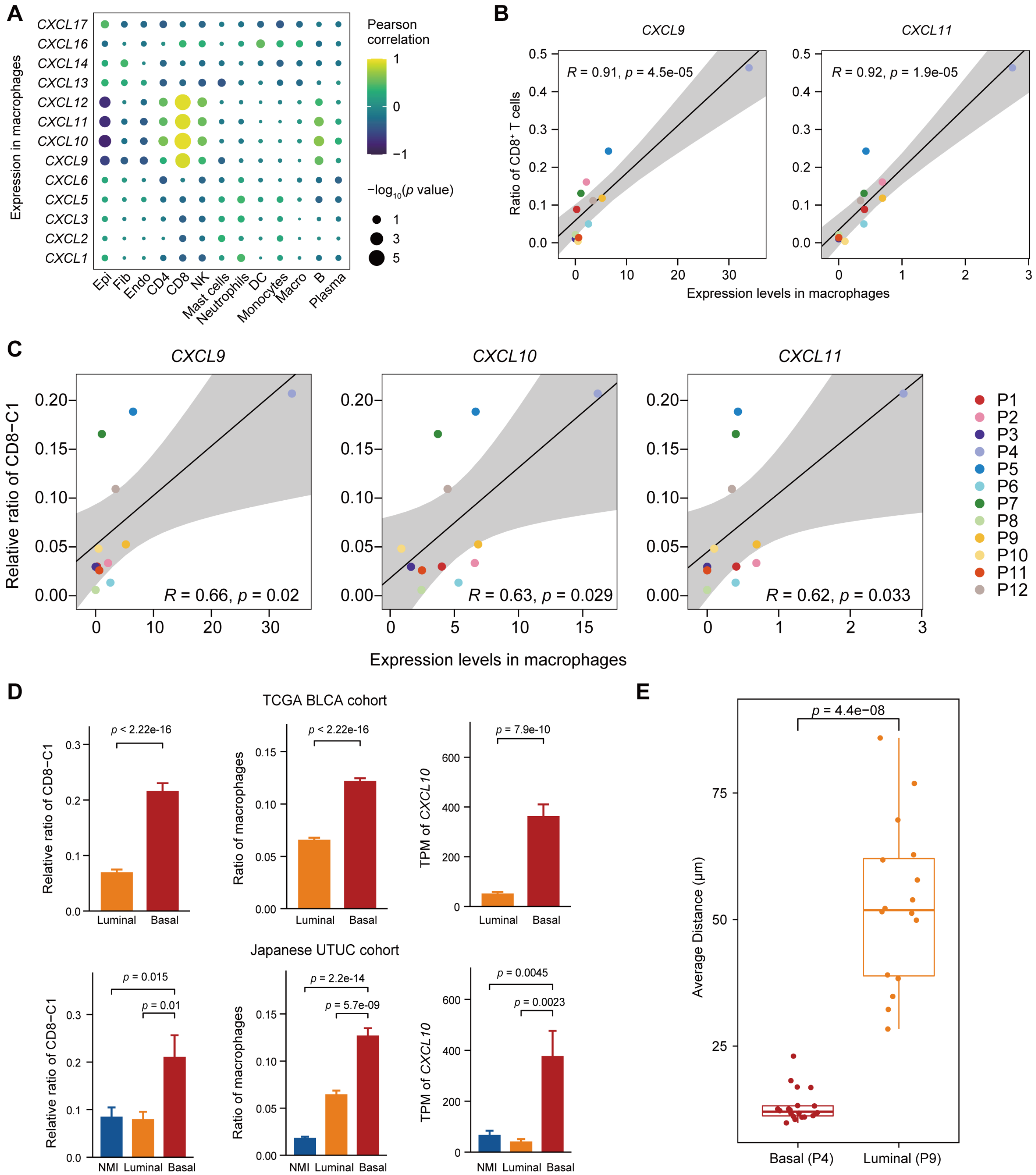

Figure S5. Cell–cell interactions between the lymphoid and myeloid compartments. Related to Figure 5

(A) Dot plot showing the correlation between the expression of *CXCL* in macrophages and the proportion of cell-types. Colors indicate Pearson correlation values (r). Size of circles shows the statistical significance.

(B and C) Scatterplot showing the correlation between the expression of *CXCL* in macrophages and the proportion of CD8^{+} T cells (B) and the relative abundance of CD8-C1 (C) in the scRNA-seq dataset. The tumor thrombus (P4TT) was excluded in the analysis. Relative ratio of CD8-C1 is defined as the proportion

of CD8-C1 in T/NK populations. Pearson coefficient (*R*) and associated *P* value are reported.

(D) Bar plots showing the relative abundance of CD8-C1, proportion of macrophages, and *CXCL10* expression for the three subtypes in the TCGA-BLCA (top) and Japanese UTUC (bottom) cohorts. Relative ratio of CD8-C1 is defined as the proportion of CD8-C1 in T/NK populations. The error bars represent mean ± standard error of the mean. *P* values were produced with *t*-test.

(E) Box plots showing the average distance between the CD68-expressing macrophages (*CXCL10*$^+$) and CD8-expressing CD8$^+$ T cells (*CXCR3*$^+$) in the basal (P4) and luminal (P9) subtype.

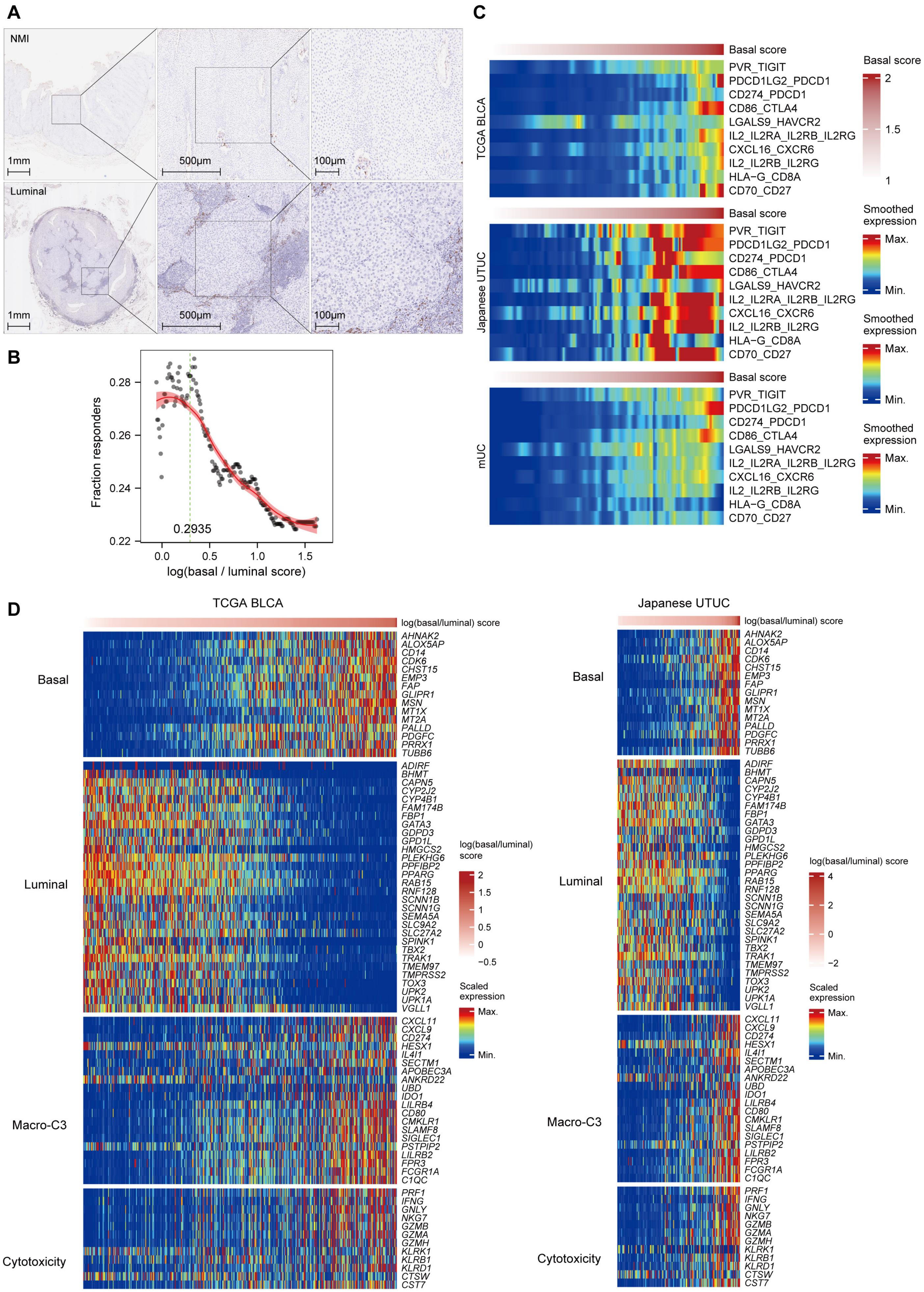
A
NMI
1mm
500μm
100μm
Luminal
1mm
500μm
100μm
B
Fraction responders
0.28
0.26
0.24
0.22
0.2935
0.0
0.5
1.0
1.5
log(basal / luminal score)
C
Basal score
TCGA BLCA
PVR_TIGIT
PDCD1LG2_PDCD1
CD274_PDCD1
CD86_CTLA4
LGALS9_HAVCR2
IL2_IL2RA_IL2RB_IL2RG
CXCL16_CXCR6
IL2_IL2RB_IL2RG
HLA−G_CD8A
CD70_CD27
Japanese UTUC
mUC
Smoothed expression
Max.
Min.
2
1.5
1
D
TCGA BLCA
Japanese UTUC
log(basal/luminal) score
Basal
AHNAK2
ALOX5AP
CD14
CDK6
CHST15
EMP3
FAP
GLIPR1
MSN
MT1X
MT2A
PALLD
PDGFC
PRRX1
TUBB6
Luminal
ADIRF
BHMT
CAPN5
CYP2J2
CYP4B1
FAM174B
FBP1
GATA3
GDPD3
GPD1L
HMGCS2
PLEKHG6
PPFIBP2
PPARG
RAB15
RNF128
SCNN1B
SCNN1G
SEMA5A
SLC9A2
SLC27A2
SPINK1
TBX2
TRAK1
TMEM97
TMPRSS2
TOX3
UPK2
UPK1A
VGLL1
Macro-C3
CXCL11
CXCL9
CD274
HESX1
IL4I1
SECTM1
APOBEC3A
ANKRD22
UBD
IDO1
LILRB4
CD80
CMKLR1
SLAMF8
SIGLEC1
PSTPIP2
LILRB2
FPR3
FCGR1A
C1QC
Cytotoxicity
PRF1
IFNG
GNLY
NKG7
GZMB
GZMA
GZMH
KLRK1
KLRB1
KLRD1
CTSW
CST7
log(basal/luminal) score
2
1.5
1
0.5
0
−0.5
4
2
0
−2
Scaled expression
Max.
Min.

Figure S6. Associations among UTUC subtypes, immune phenotypes, and the response to PD-L1 blockade.

(A) IHC staining of CD8 indicating the status of $CD8^+$ T cell infiltration (brown) in luminal (P2) and NMI (P3) UTUC samples. Scale bars correspond to 1mm, 500 μm and 100 μm in the large and small panels, respectively.

(B) Scatter plot showing ICI response rate decreased when patients with higher log(basal/luminal) scores were considered in the IMvigor210 cohort.

(C) Heatmap showing the strength of the indicated interactions in the TCGA BLCA, Japanese UTUC and IMvigor210 cohorts. The selected interactions are receptor-ligand pairs expressed by macrophages and $CD8^+$ T cells and are most positively associated with the percentage of CD8-C1 cells (exhausted $CD8^+$ T cells) in the T/NK population of the scRNA dataset. Colors represent average gene expression levels of ligand-receptor pairs that were smoothed with locally-weighted polynomial regression and interpolated at evenly distributed log(basal/luminal) score.

(D) Heatmaps indicating the scaled expression of selected genes from different categories in the TCGA BLCA and Japanese UTUC cohorts. The samples were ordered by log(basal/luminal) score.

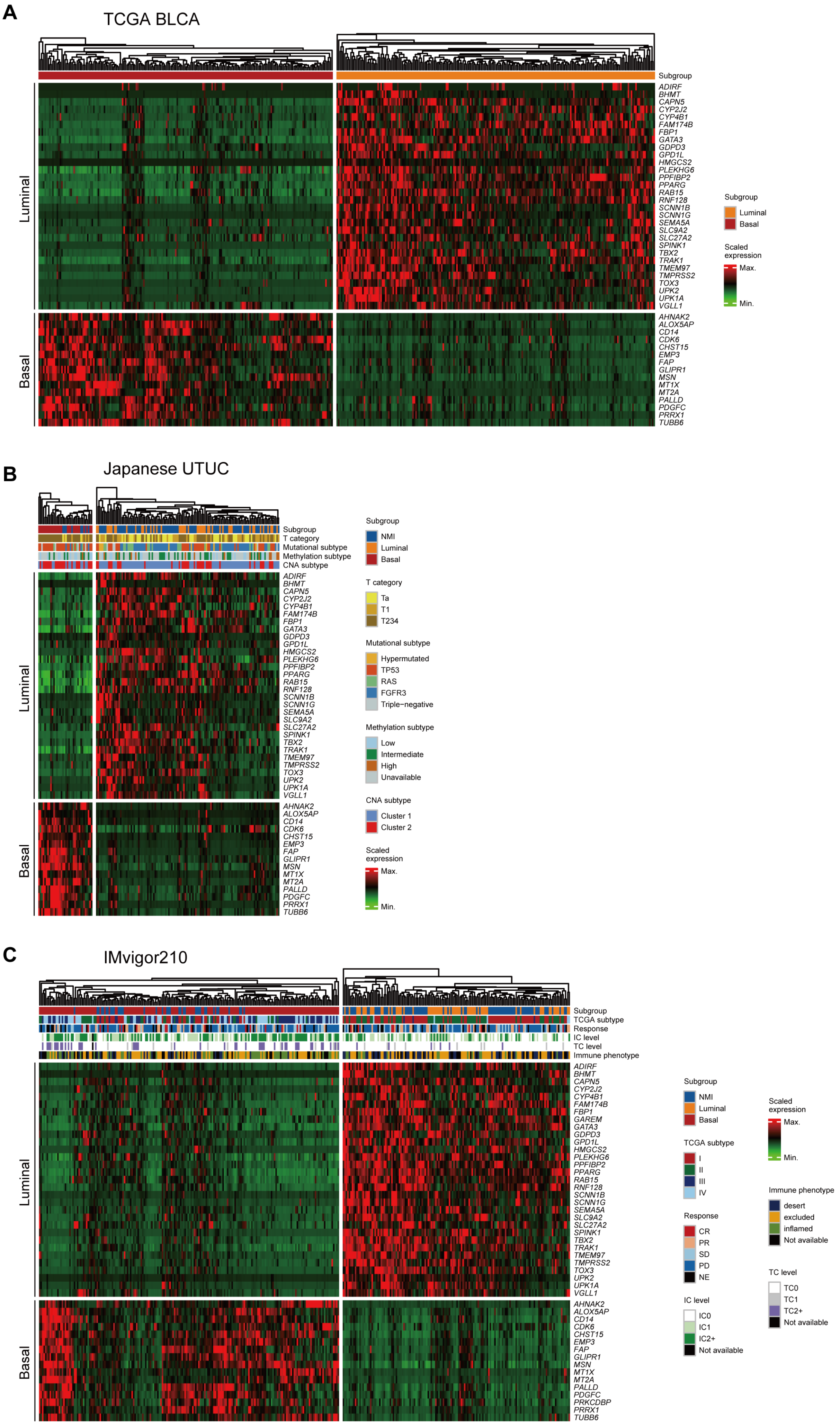
A
TCGA BLCA
Subgroup
Luminal
Basal
ADIRF
BHMT
CAPN5
CYP2J2
CYP4B1
FAM174B
FBP1
GATA3
GDPD3
GPD1L
HMGCS2
PLEKHG6
PPFIBP2
PPARG
RAB15
RNF128
SCNN1B
SCNN1G
SEMA5A
SLC9A2
SLC27A2
SPINK1
TBX2
TRAK1
TMEM97
TMPRSS2
TOX3
UPK2
UPK1A
VGLL1
AHNAK2
ALOX5AP
CD14
CDK6
CHST15
EMP3
FAP
GLIPR1
MSN
MT1X
MT2A
PALLD
PDGFC
PRRX1
TUBB6
Subgroup
Luminal
Basal
Scaled expression
Max.
Min.
B
Japanese UTUC
Subgroup
T category
Mutational subtype
Methylation subtype
CNA subtype
Luminal
Basal
Subgroup
NMI
Luminal
Basal
T category
Ta
T1
T234
Mutational subtype
Hypermutated
TP53
RAS
FGFR3
Triple−negative
Methylation subtype
Low
Intermediate
High
Unavailable
CNA subtype
Cluster 1
Cluster 2
Scaled expression
Max.
Min.
C
IMvigor210
Subgroup
TCGA subtype
Response
IC level
TC level
Immune phenotype
Luminal
Basal
GAREM
PRKCDBP
Subgroup
NMI
Luminal
Basal
TCGA subtype
I
II
III
IV
Response
CR
PR
SD
PD
NE
IC level
IC0
IC1
IC2+
Not available
Scaled expression
Max.
Min.
Immune phenotype
desert
excluded
inflamed
Not available
TC level
TC0
TC1
TC2+
Not available

Figure S7. Clustering analysis of RNA-seq data from public datasets.

(A-C) The clustering was based on the scaled expression of the BASE47 gene set (rows, $K = 2$). Three public RNA-seq datasets were used: TCGA-BLCA (n = 404), Japanese UTUC (n = 158), and the IMvigor210 cohort (n = 348). Subtype classification and available clinical information as well as molecular features for each sample are indicated in heatmap annotations.

1. Young MD, Behjati S. SoupX removes ambient RNA contamination from droplet-based single-cell RNA sequencing data. Gigascience. 2020; 9.

2. Germain PL, Sonrel A, Robinson MD. pipeComp, a general framework for the evaluation of computational pipelines, reveals performant single cell RNA-seq preprocessing tools. Genome Biol. 2020; 21: 227.

3. Baslan T, Kendall J, Rodgers L, Cox H, Riggs M, Stepansky A, et al. Genome-wide copy number analysis of single cells. Nat Protoc. 2012; 7: 1024-41.

4. Adalsteinsson VA, Ha G, Freeman SS, Choudhury AD, Stover DG, Parsons HA, et al. Scalable whole-exome sequencing of cell-free DNA reveals high concordance with metastatic tumors. Nat Commun. 2017; 8: 1324.

5. Robinson JT, Thorvaldsdottir H, Winckler W, Guttman M, Lander ES, Getz G, et al. Integrative genomics viewer. Nat Biotechnol. 2011; 29: 24-6.

6. Cancer Genome Atlas Research N. Comprehensive molecular characterization of urothelial bladder carcinoma. Nature. 2014; 507: 315-22.

7. Fujii Y, Sato Y, Suzuki H, Kakiuchi N, Yoshizato T, Lenis AT, et al. Molecular classification and diagnostics of upper urinary tract urothelial carcinoma. Cancer Cell. 2021; 39: 793-809 e8.

8. Mariathasan S, Turley SJ, Nickles D, Castiglioni A, Yuen K, Wang Y, et al. TGFbeta attenuates tumour response to PD-L1 blockade by contributing to exclusion of T cells. Nature. 2018; 554: 544-8.

9. Colaprico A, Silva TC, Olsen C, Garofano L, Cava C, Garolini D, et al. TCGAbiolinks: an R/Bioconductor package for integrative analysis of TCGA data. Nucleic Acids Res. 2016; 44: e71.

10. Liu J, Lichtenberg T, Hoadley KA, Poisson LM, Lazar AJ, Cherniack AD, et al. An Integrated TCGA Pan-Cancer Clinical Data Resource to Drive High-Quality Survival Outcome Analytics. Cell. 2018; 173: 400-16 e11.

11. Bi K, He MX, Bakouny Z, Kanodia A, Napolitano S, Wu J, et al. Tumor and immune reprogramming during immunotherapy in advanced renal cell carcinoma. Cancer Cell. 2021; 39: 649-61 e5.

12. Damrauer JS, Hoadley KA, Chism DD, Fan C, Tiganelli CJ, Wobker SE, et al. Intrinsic subtypes of high-grade bladder cancer reflect the hallmarks of breast cancer biology. Proc Natl Acad Sci U S A. 2014; 111: 3110-5.

13. Jew B, Alvarez M, Rahmani E, Miao Z, Ko A, Garske KM, et al. Accurate estimation of cell composition in bulk expression through robust integration of single-cell information. Nat Commun. 2020; 11: 1971.
